# SPSP-1 is a sperm protein required for *C. elegans* hermaphrodite sperm production and embryogenesis

**DOI:** 10.1242/bio.062634

**Published:** 2026-06-30

**Authors:** Darline Murat, Margaret Nagorski, Ji Kent Kwah, Shannon Pfeiffer, Nancy Duker, Frank Döring, Aimee Jaramillo-Lambert

**Affiliations:** ^1^ Department of Biological Sciences, University of Delaware, Newark, Delaware 19716, USA; ^2^ Department of Molecular Prevention, University of Kiel, 24106 Kiel, Germany

**Keywords:** Sperm, Spermatogenesis, Spermiogenesis, Fertilization, *C. elegans*, SPE-11

## Abstract

Development requires the fusion of two gametes each with a haploid complement of DNA and the necessary components (RNA, proteins, organelles) needed for early cellular processes and divisions. The *C. elegans* paternal-effect embryonic lethal gene, *spe-11*, is one of the few known sperm components required for normal development. Sperm-provided SPE-11 forms a complex with the oocyte protein OOPS-1 at fertilization that mediates egg activation events. In this manuscript, we identified SPSP-1, a sperm protein that is associated with SPE-11. We found that SPSP-1 co-localizes with SPE-11 during spermatogenesis and in spermatids. SPSP-1 does not appear to play a role in egg activation like SPE-11. However, SPSP-1 is required to produce normal levels of hermaphrodite sperm and for wild-type levels of embryonic viability.

## Teaser

**Summary:** A newly identified sperm protein, SPSP-1, is required for hermaphrodite fertility and embryonic viability.

## INTRODUCTION

Oocytes and sperm are highly specialized cells that differ not only in their morphologies, but also in their contributions to fertilization and embryogenesis. During fertilization, a single sperm binds and fuses with an oocyte, triggering egg activation and initiating a series of cellular changes that transitions the quiescent oocyte into a totipotent, developmentally active embryo. Egg activation is composed of several events including an increase in intracellular calcium, resumption and completion of oocyte meiotic divisions, cytoskeletal rearrangements, and modification of the outer covering of the oocyte to prevent polyspermy and to support zygotic development (Horner and Wolfner, 2008; Krauchunas and Wolfner, 2013).

Each gamete provides components necessary for development. Oocytes are large, sedentary cells that provide a haploid genome and large stockpiles of RNA and proteins necessary for early embryonic cell divisions until zygotic transcription is initiated. Sperm are small, motile cells that provide a haploid genome, as well as centrosomes and other necessary molecules, including the signal for the initiation of egg activation (Horner and Wolfner, 2008; Jaramillo-Lambert and Krauchunas, 2025). Although important, the molecular details of the sperm's contribution to early embryogenesis remain largely unknown.

In *C. elegans*, SPE-11 is a sperm-specific protein that plays a crucial role in egg activation and is currently the only known strictly paternal effect embryonic lethal (PEL) gene in *C. elegans*. *spe-11* mutant sperm are capable of fertilization, but the resulting embryos are inviable (L'Hernault et al., 1988). Mutant embryos fertilized by *spe-11* sperm have defects in multiple early developmental and egg activation events, including oocyte meiotic cytokinesis failure, eggshell integrity defects, and failure to prevent polyspermy (Browning and Strome, 1996; Hill et al., 1989; Johnston et al., 2010). SPE-11 is only expressed during spermatogenesis of both males and hermaphrodites. Starting in late prophase SPE-11 localizes as distinct puncta before forming a ring of puncta around the condensed DNA of post-meiotic sperm (Browning and Strome, 1996; Jaramillo-Lambert and Golden, 2020). Recently, we along with our collaborators discovered that SPE-11 forms a complex with OOPS-1, an oocyte-specific protein. An OOPS-1-SPE-11 complex forms upon fertilization and this complex is required to regulate egg activation events, including the completion of oocyte meiosis, eggshell synthesis, and the block to polyspermy (Tsukamoto et al., 2025). In work from Tsukamoto et al., two additional sperm-specific proteins, GSP-3 and GSP-4 (PPI phosphatase subunits), were found to interact with both OOPS-1 and SPE-11. While *gsp-3 gsp-4* double mutants have sperm defects, *spe-11* does not appear to be required for spermatogenesis (Browning and Strome, 1996; Wu et al., 2012). Rather, the OOPS-1-SPE-11 complex interactions with GSP-3/4 are most likely required post-fertilization in the embryo (Ataeian et al., 2016; Tsukamoto et al., 2025). While these recent discoveries have identified some of the molecular interactions of SPE-11 in fertilized oocytes, it is likely that additional oocyte proteins interact with SPE-11, and spermatogenesis/sperm-specific proteins that interact with SPE-11 have yet to be identified.

In this manuscript, we identified SPSP-1 (sperm partner of SPE-11), a spermatogenesis protein that is associated with SPE-11. We found that SPSP-1 co-localizes with SPE-11 during both hermaphrodite and male spermatogenesis. Hermaphrodite *spsp-1* null mutants show reduced levels of sperm and a reduction in embryonic viability. Lastly, we show that SPSP-1 and SPE-11 are reciprocally required for localization to late prophase nuclei and spermatids.

## RESULTS

### *spsp-1* mutants are subfertile

**Fig. 1.**
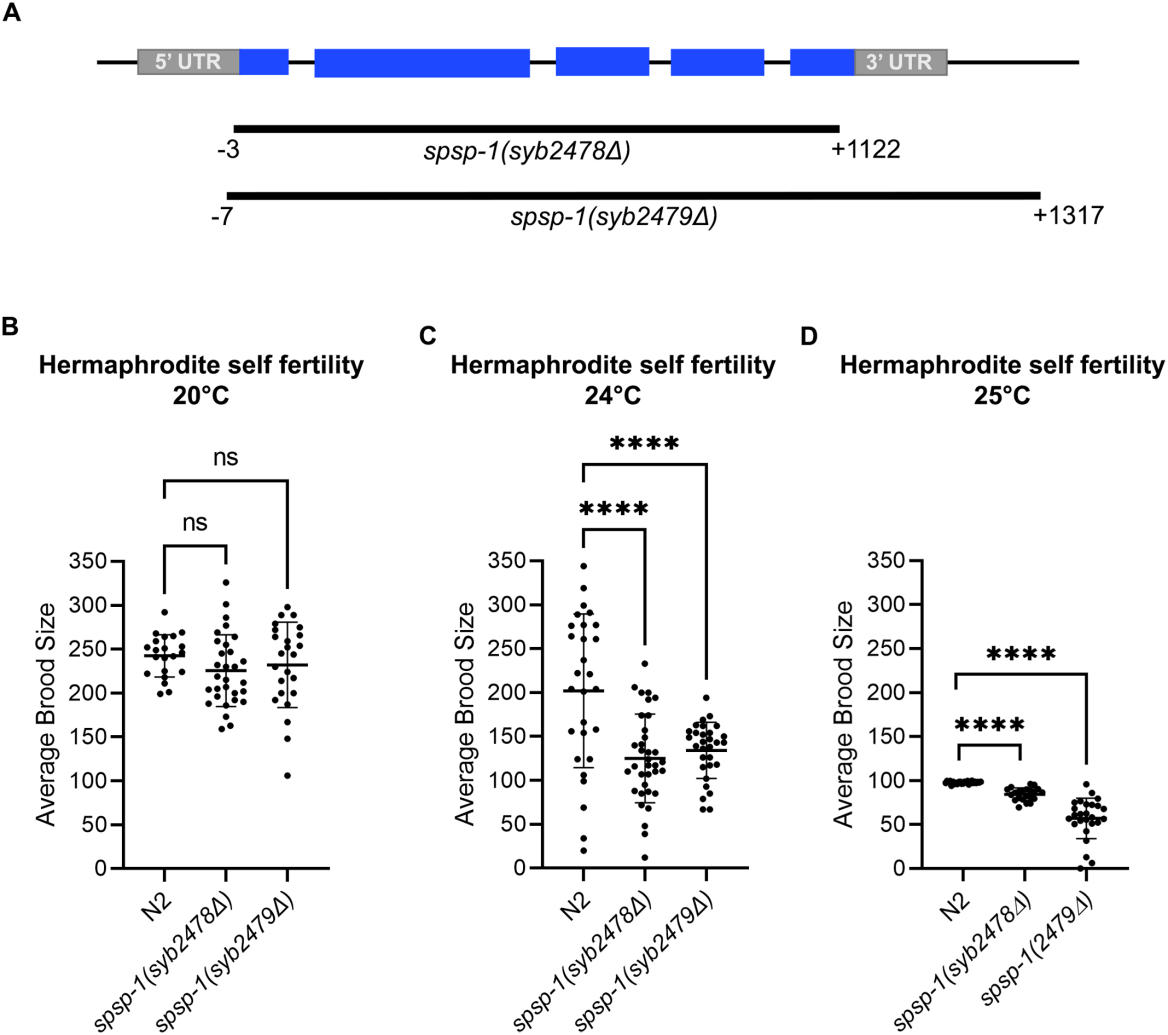
***spsp-1* mutations result in decreased brood sizes.** (A) Diagram of the *spsp-1* gene. *spsp-1* contains 1289 bp with five exons. *spsp-1(syb2478Δ)* is a 1125 bp deletion and *spsp-1(syb2479Δ)* is a 1324 bp deletion spanning into the 3′ UTR. (B-D). Average brood sizes of N2 and *spsp-1Δ* mutants at 20°C, 24°C, and 25°C. Sample sizes (number of individual hermaphrodite broods) at 20°C: N2 *N*=21, *spsp-1(syb2478Δ) N*=30, and *spsp-1(syb2479Δ) N*=24; 24°C N2 *N*=28, *spsp-1(syb2478Δ) N*=35, and *spsp-1(syb2479Δ) N*=30; 25°C N2 *N*=25 *spsp-1(syb2478Δ) N*=22, and *spsp-1(syb2479Δ) N*=26. Bars represent mean±s.d. Statistics were performed using a Welch's *t*-test. ns, not significant, ****P<0.0001.

To identify the genetic pathways in which *spe-11* is involved, a yeast two-hybrid screen was performed using *spe-11* cDNA as bait (Hybrigenics Corporation, Boston, MA, USA). The uncharacterized open reading frame F07A5.2 was identified as a strong candidate interacting partner. FPKM expression values across *C. elegans* life stages (PolyA+modENCODE library) show highest expression of F07A5.2 in males and L4 hermaphrodites suggesting likely sperm expression. Therefore, F07A5.2 was renamed as sperm partner of SPe-11 (*spsp-1*). To understand the function of *spsp-1*, we assessed two deletion alleles (*syb2478* and *syb2479*), which remove exons one through five and one through five plus the 3′UTR, respectively (Fig. 1A), for reproductive capacity and embryonic viability. Unmated *spsp-1(syb2478*Δ*)* and *spsp-1(syb2479*Δ*)* hermaphrodites produced normal levels of progeny compared to wild-type (N2) hermaphrodites at 20°C (Fig. 1B) [N2=242.4±24.1, *spsp-1(syb2478*Δ*)*=225.6±40.9, *spsp-1(syb2479*Δ*)*=232.1±48.5]. However, at elevated culture temperatures both *spsp-1(syb2478*Δ*)* and *spsp-1(syb2479*Δ*)* were subfertile (Fig. 1C,D) [24°C: N2=202.0±87.6, *spsp-1(syb2478*Δ*)*=125.1±50.4, *spsp-1(syb2479*Δ*)*=134.1±331.9; 25°C: N2=97.9±1.5, *spsp-1(syb2478*Δ*)*=84.6±7.1, *spsp-1(syb2479*Δ*)*=56.9±23.1]. We also found that the *spsp-1* mutations showed a subtle temperature-sensitive embryonic lethal phenotype (Fig. S1A-C) [20°C: N2=97.7%±0.58, *spsp-1(syb2478*Δ*)*=88.2%±1.63, *spsp-1(syb2479*Δ*)*=94.5%±2.0; 24°C: N2=94.0%±2.68, *spsp-1(syb2478*Δ*)*=73.7%±3.48, *spsp-1(syb2479*Δ*)*=78.6%±0.38; 25°C: N2=97.9%±0.41, *spsp-1(syb2478*Δ*)*=85.1%±1.60, *spsp-1(syb2479*Δ*)*=58.1%±5.10]. As the *spsp-1* fertility phenotypes are temperature dependent, all subsequent experiments were performed at 24°C.

To determine the developmental stage the dead embryos reached, we imaged embryos by DIC and DAPI staining. As only ∼20% of *spsp-1* mutant embryos are nonviable, we recovered dead embryos from plates incubated at 24°C for 48 h. Embryos from both *spsp-1(syb2478*Δ*)* and *spsp-1(syb2479*Δ*)* were able to undergo several rounds of cell division. Most of the embryos appeared to have arrested during gastrulation (Fig. S1D). Some embryos had creases that resemble attempted folding of the embryo during the elongation stage (Fig. S1D, example 2). However, these embryos do not resemble the characteristic morphologies of the elongation stage (e.g. comma, 1.5-fold, 2-fold, or 3-fold) (Hall et al., 2017). From these observations we conclude that *spsp-1(syb2478*Δ*)* and *spsp-1(syb2479*Δ*)* embryos arrest between gastrulation and elongation, suggesting that SPSP-1 is required for embryonic development post gastrulation.

**Fig. 2.**
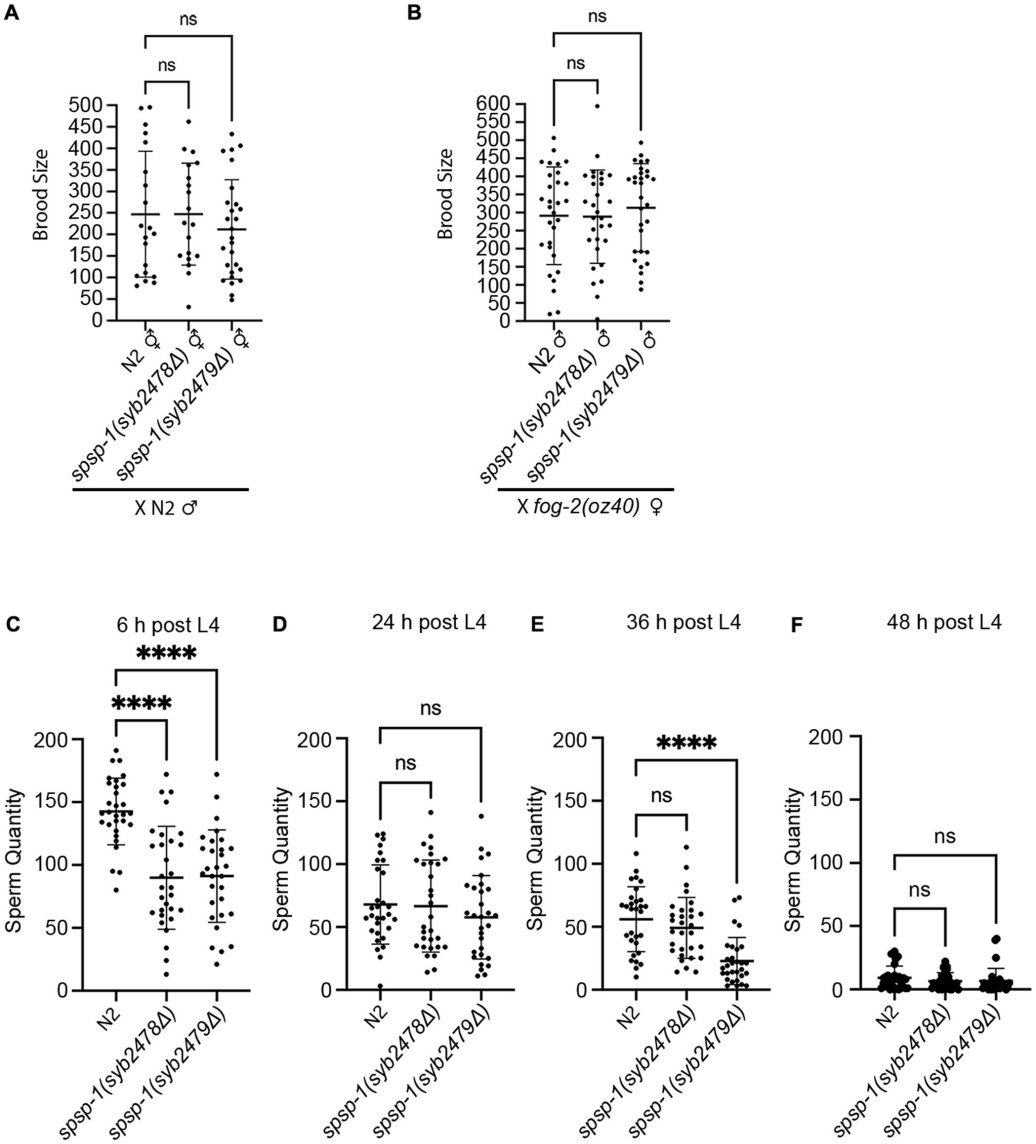
***spsp-1* mutant hermaphrodites produce fewer sperm.** (A) Wild-type sperm rescues the reduced brood sizes of *spsp-1Δ* mutants. Brood sizes of N2 males crossed with *spsp-1Δ* mutant hermaphrodites at 24°C. N2 *N*=20, *spsp-1(syb2478Δ) N*=19, and *spsp- 1(syb2479Δ) N*=27. Bars represent mean±s.d. One-way ANOVA. ns, not significant. (B) *spsp-1Δ* males produce normal brood sizes when mated to wild-type females. Brood sizes of *spsp-1Δ* males crossed with the feminized *fog-2(oz40)* animals at 24°C. N2 *N*=30, *spsp-1(syb2478Δ) N*=30, and *spsp-1(syb2479Δ) N*=30. One-way ANOVA. ns, not significant. (C-F) Number of sperm per individual spermathecae of adult hermaphrodites 6 h (C), 24 h (D), 36 h (E), and 48 h (F) post L4. N2 *N*=30, *spsp-1(syb2478Δ) N*=30, and *spsp-1(syb2479Δ) N*=30 for all time points. Bars represent mean±s.d. One-way ANOVA; ****P<0.0001.

Next, we sought to determine if the reduced brood sizes of the *spsp-1* mutants were a consequence of defective oocytes, defective sperm, or a combination of both through reciprocal mutant crosses. First, *spsp-1(syb2478*Δ*)* and *spsp-1(syb2479*Δ*)* hermaphrodites were mated with N2 (wild-type) males at 24°C. Mutant hermaphrodites can produce progeny with brood sizes comparable to N2 hermaphrodites mated with N2 males [Fig. 2A, N2=247.1±146.1, *spsp-1(syb2478)*=247.2±118.3, *spsp-1(syb2479)*=212.1±115.4], suggesting *spsp-1* oocytes are fertilization competent. To evaluate male fertility, *spsp-1(syb2478*Δ*)* and *spsp-1(syb2479*Δ*)* males were mated with *fog-2(oz40)* worms. *fog-2(oz40)* mutants cannot produce self-sperm; thus, they are essentially female and any progeny produced by *fog-2(oz40)* would be sired by males. Control males produced broods of ∼291.1 progeny with both *spsp-1* mutant males producing similar brood sizes [Fig. 2B, *spsp-1(syb2478)*=288.6±129.0, *spsp-1(syb2479)*=313.3±121.8].

We also examined the progeny of the reciprocal matings for embryonic lethality. The slight reduction in embryonic viability of the *spsp-1* mutants was restored when wild-type sperm were used for fertilization [Fig. S2, N2=96.5%±0.6, *spsp-1(syb2478)*=94.6±1.2, *spsp-1(syb2479)*=95.4±1.0]. These results suggest that the reduction in brood and embryonic viability could stem from a sperm defect. When *spsp-1* mutant males were crossed to *fog-2(oz40)* control females, embryonic viability was very slightly, yet significantly reduced when *spsp-1(syb2478)* mutants were the sires [Fig. S2B, N2=98.6%±0.2, *spsp-1(syb2478)*=96.1%±0.5]. There was not a statistically significant difference in embryonic viability between N2 and *spsp-1(syb2479)* sires [Fig. S2B, N2=98.6%±0.2, *spsp-1(syb2479)*=98.3%±0.3]. These results indicate that *spsp-1* is not required for male fertility. However, *spsp-1* is required for full hermaphrodite self-fertility.

### *spsp-1* mutant hermaphrodites produce fewer sperm

As both *spsp-1(syb2478*Δ*)* and *spsp-1(syb2479*Δ*)* self-fertilizing hermaphrodites produce fewer overall numbers of progeny compared to wild-type hermaphrodites, we next sought to determine if there were changes in sperm or oocyte morphology as well as fertilization competency. Sperm were found within the spermathecae (the site of fertilization) of wild-type and mutant hermaphrodites (Geldziler et al., 2011; Ward and Carrel, 1979). However, quantification of the number of sperm before the first ovulation (6 h post-L4) showed that *spsp-1* mutant hermaphrodites produce a reduced number of sperm compared to wild type [Fig. 2C, N2=142.6±26.5, *spsp-1(syb2478)*=89.9±40.9, *spsp-1(syb2479)*=91.2±36.8]. Quantification of the number of sperm over the course of 2 days showed similar numbers of sperm at the 24 h and 48 h time points (Fig. 2D,F). However, there were significantly fewer sperm in the spermathecae of *spsp-1(syb2479)* at 36 h (22.7±18.6, Fig. 2E).

**Fig. 3.**
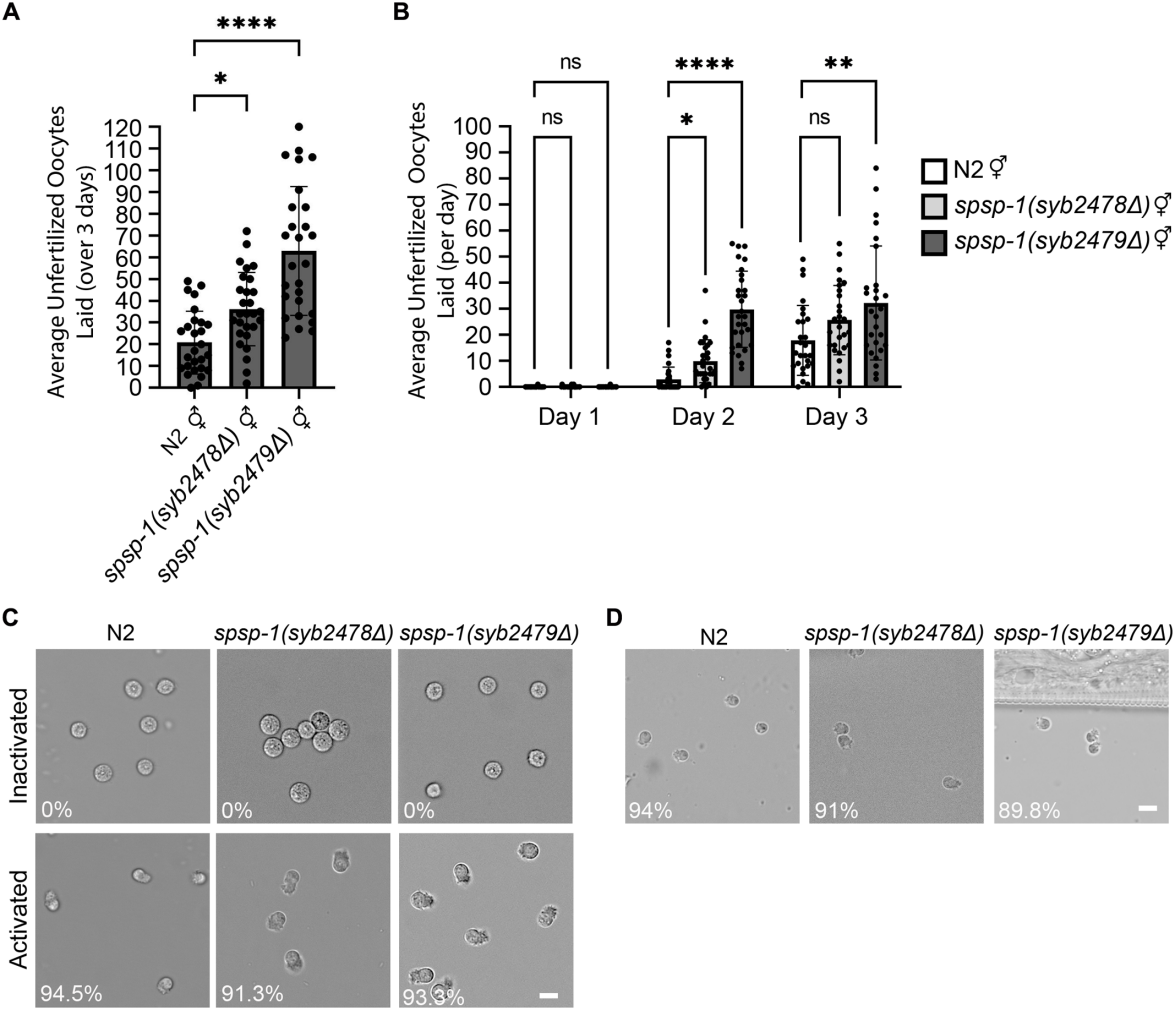
***spsp-1* mutants lay unfertilized oocytes.** (A) The number of unfertilized oocytes of N2 and *spsp-1Δ* hermaphrodites at 24°C presented as an average of the 72-h assay window. Sample sizes: N2 *N*=27, *spsp-1(syb2478Δ) N*=28, and *spsp-1(syb2479Δ) N*=27. Bars represent mean±s.d. One-way ANOVA, *P<0.05, ****P<0.0001. (B) The average number of unfertilized oocytes of N2 and *spsp-1Δ* mutant hermaphrodites at 24°C by day. Bars represent mean±s.d. Two-way ANOVA; ns, not significant, *P<0.05, **P<0.02, ****P<0.0001. (C) *In vitro* pronase-activated N2 and *spsp-1Δ* male spermatozoa. Sperm sample sizes (inactivated): N2 *N*=139, *spsp-1(syb2478Δ) N*=125, and *spsp-1(syb2479Δ) N*=102. Sperm sample sizes (activated): N2 *N*=128, *spsp-1(syb2478Δ) N*=129, and *spsp-1(syb2479Δ) N*=104. Scale bar: 5 μm. (D) Hermaphrodite self-sperm activated *in vivo*. Sperm sample sizes: N2 *N*=149, *spsp-1(syb2478Δ) N*=100, and *spsp-1(syb2479Δ) N*=49. Scale bar: 5 μm. The percentage of activated sperm is indicated in the bottom left corner. Sperm activation data was collected from at least three replicates.

In *C. elegans*, sperm secrete a hormone-like signal that stimulates oocyte maturation and ovulation (Miller et al., 2001). During the course of the brood sizing assays, we observed that *spsp-1* mutants seemed to lay more unfertilized oocytes than wild-type controls. Therefore, the number of oocytes laid by the *spsp-1(syb2478Δ)* and *spsp-1(syb2479Δ)* hermaphrodites at 24°C was quantified over a period of 72 h. On average both deletion mutants laid increased numbers of oocytes when compared to the wild-type control [Fig. 3A, N2=20.8±14.4, *spsp-1(syb2478)*=36.2±16.9, *spsp-1(syb2479)*=63.0±29.6]. When the data were analyzed by day, both deletion mutants laid an increased number of oocytes compared to the control on day two and three of the experiment (Fig. 3B). To determine if the increased number of oocytes laid by the *spsp-1* mutant hermaphrodites correlated with the decreased brood size of the mutants, we analyzed this same data set for the average number of progeny produced and embryonic viability of the progeny. The brood size over a 72-h period reflected the same phenotypes we observed in Fig. 1; both *spsp-1(syb2478)* and *spsp-1(syb2479)* had reduced brood sizes compared to N2 [Fig. S3A, N2=228.8±10.0, *spsp-1(syb2478)*=186.1±8.5, *spsp-1(syb2479)*=176.5±6.9]. When the brood sizes were analyzed by day, there was not a significant difference in the number of progeny on day one, but a reduced number of progeny were observed on day two and three (Fig. S3B). These data are consistent with the reduced number of hermaphrodite self-sperm produced by *spsp-1(syb2478)* and *spsp-1(syb2479)* (Fig. 2C). As the hermaphrodite self-sperm are depleted through fertilization, over time, fewer progeny are produced.

As we also found that *spsp-1(syb2478)* and *spsp-1(syb2479)* had a subtle decrease in embryonic viability, we asked if the increased number of oocytes laid correlated with embryonic viability. Embryonic viability over the 72-h period was reduced for both *spsp-1(syb2478)* and *spsp-1(syb2479)* compared to N2 [Fig. S3C, N2=97.0%±1.1., *spsp-1(syb2478)*=86.9%±3.2, *spsp-1(syb2479)*=80.4%± 2.5]. The percentage of embryonic viability by day showed that embryonic viability was reduced for each day (Fig. S3D). As dead embryos were observed each day with the numbers proportional to the number of embryos laid per day, we concluded that the defects causing embryonic lethality remain consistent over the reproductive lifespan of the hermaphrodite.

We next asked if *spsp-1* mutant sperm exhibited any changes in morphology or activation. Meiosis produces round haploid spermatids that must then differentiate (spermiogenesis or sperm activation) into motile spermatozoa (Chu and Shakes, 2013). In *C. elegans* males, spermatids activate when they mix with the seminal fluid during ejaculation (Hirsh et al., 1976; Klass et al., 1976; Ward and Carrel, 1979). Sperm activation can also be induced *in vitro* using a pharmacological approach (Shakes and Ward, 1989). Male derived *spsp-1* sperm activated normally *in vitro* (Fig. 3C). In hermaphrodites, spermatids activate when they are pushed into the spermatheca at the first ovulation (Ward and Carrel, 1979). Spermatozoa dissected from the spermathecae of adult *spsp-1* mutant hermaphrodites were also activated *in vivo* (Fig. 3D). Thus *spsp-1* is not required for sperm activation in either males or hermaphrodites.

### SPSP-1 colocalizes with SPE-11 in the spermatogenic germ line

**Fig. 4.**
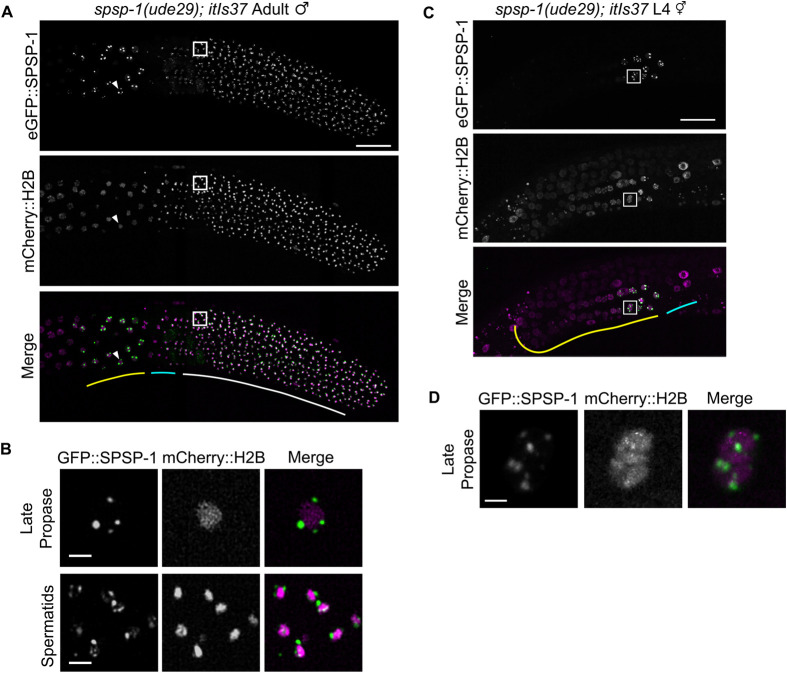
**SPSP-1 localizes to germline nuclei during spermatogenesis and post-meiotic sperm.** SPSP-1 localizes as perinuclear puncta in late meiotic prophase during spermatogenesis and in post-meiotic sperm in male and L4 hermaphrodite germlines. Live images of eGFP::SPSP-1 (green) and mCherry::H2B (magenta) localization in *spsp-1(ude29)* [*egfp::spsp-1*] I; *itIs37* [pAA64: *pie-1p::mCherry::his-58*+*unc-119(+)*] males (A) and hermaphrodites (C). Yellow lines indicate late prophase nuclei, blue lines indicate karyosome nuclei, and white lines indicate post-meiotic spermatids. Scale bar: 20 μm. (B) Zoomed in images of a late prophase nucleus (arrowhead in A) and post-meiotic sperm (white square in A) in a male germline. Scale bar: 5 μm. (D) Zoomed in images of a late prophase nucleus (white square in C) in a L4 hermaphrodite germline. Scale bar: 5 μm. Male germlines *N*=29, L4 hermaphrodite germlines *N*=29.

To investigate the localization of SPSP-1, a GFP-tagged SPSP-1 line [*spsp-1(ude29)*] was created. SPSP-1 localized to the germ lines of adult males and L4 hermaphrodites undergoing spermatogenesis. SPSP-1 first appears in late meiotic prophase and is present in post-meiotic sperm (Fig. 4A,C). In both late prophase and post-meiotic sperm SPSP-1 localized as distinct puncta around the DNA (Fig. 4B,D).

**Fig. 5.**
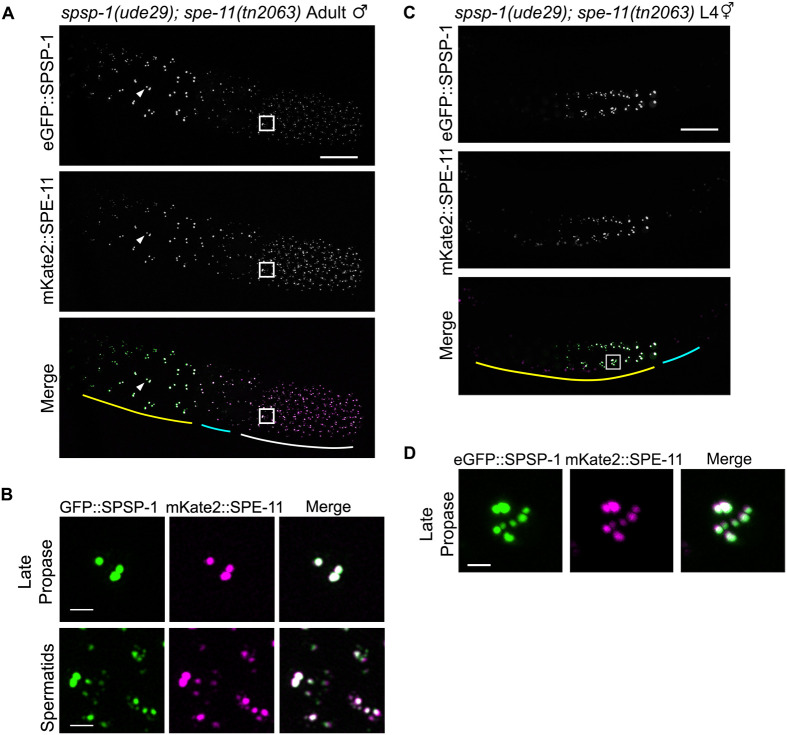
**SPSP-1 and SPE-11 colocalize during spermatogenesis.** SPSP-1 and SPE-11 colocalize as puncta in late meiotic prophase and in post-meiotic sperm of male and L4 hermaphrodite germlines. Live images of eGFP::SPSP-1 (green) and mKate2::SPE- 11 (magenta) localization in *spsp-1(ude29) spe-11(tn2063)* males (A) and L4 hermaphrodites (C). Yellow lines indicate late prophase nuclei, blue lines indicate karyosome nuclei, and white lines indicate post-meiotic spermatids. Scale bar: 20 μm. (B) Zoomed in images of a late prophase nucleus (arrowhead in A) and post-meiotic sperm (white square in A) in a male germline. Scale bar: 5 μm. (D) Zoomed in images of a late prophase nucleus (white square in C) in a L4 hermaphrodite germline. Scale bar: 5 μm. Male germlines *N*=26, L4 hermaphrodite germlines *N*=20.

The localization pattern of SPSP-1 strongly resembled the localization pattern of SPE-11 (Browning and Strome, 1996; Jaramillo-Lambert and Golden, 2020). To determine if SPSP-1 and SPE-11 colocalize, we used CRISPR to endogenously tag *spsp-1* in a strain carrying an endogenously tagged *spe-11* [*egfp::spsp-1(ude29) mkate2::spe-11(tn2063)*]. Live imaging of both male and L4 hermaphrodite germ lines showed that SPE-11 and SPSP-1 colocalize as perinuclear puncta around late prophase and post-meiotic spermatids (Fig. 5A-D).

### SPSP-1 and SPE-11 are reciprocally required for proper localization in spermatids

**Fig. 6.**
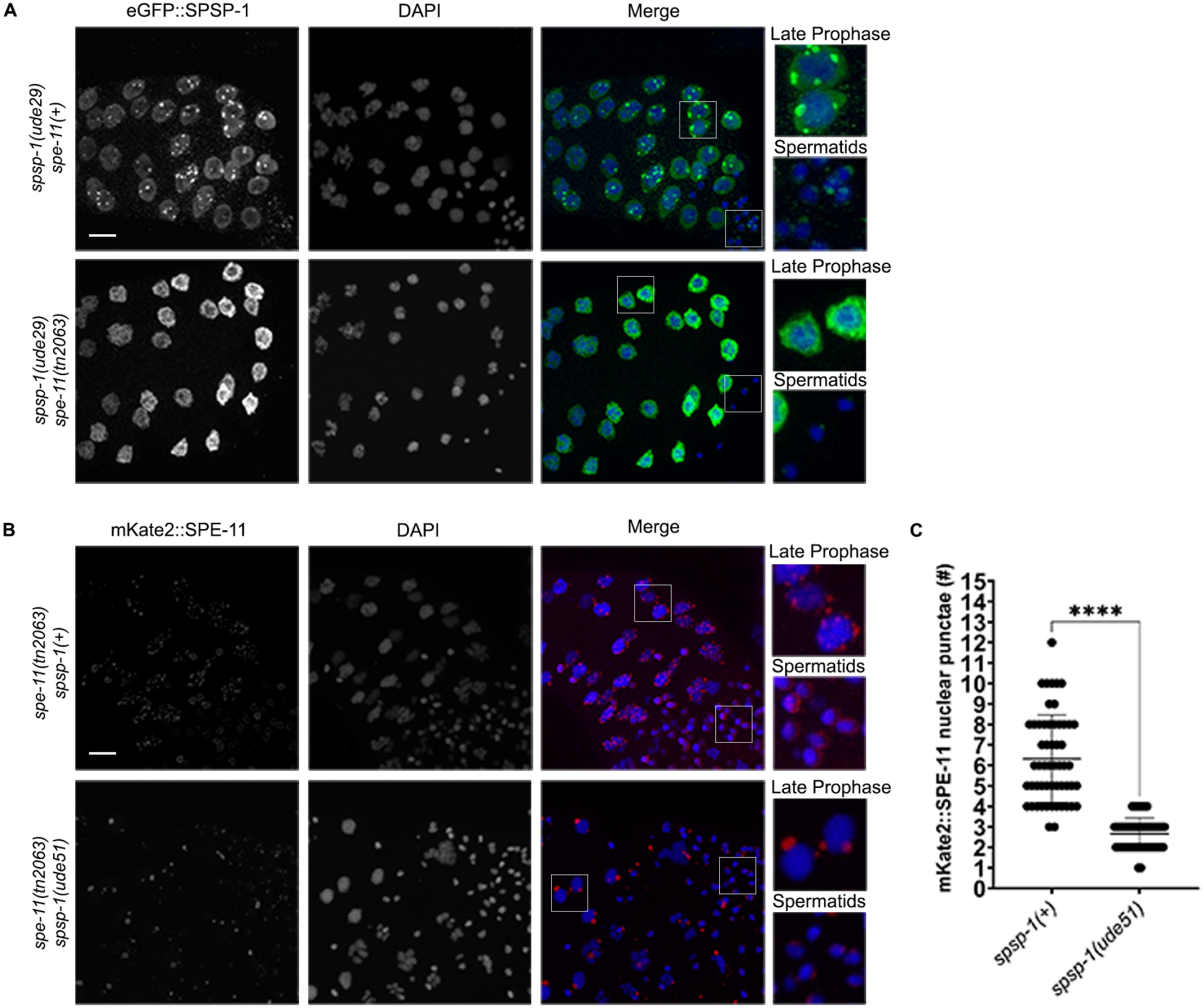
**SPE-11 and SPSP-1 are co-dependent for localization.** (A) Immunostaining of eGFP::SPSP-1 in late meiotic prophase and post-meiotic sperm of control [*spsp-1(ude39) spe-11(+)*] and *spsp-1(ude29) spe-11(tn2059*Δ*)* male germ lines. (B) Immunostaining of mKate2::SPE-11in late meiotic prophase and post-meiotic sperm of control [*spsp-1(+) spe-11(tn2063)*] and *spsp-1(ude51*Δ*) spe-11(tn2063)* male germ lines. Scale bar: 5 μm (A,B). (C) Quantification of mKate2::SPE-11 puncta in late prophase nuclei. Sample sizes: *spsp-1(ude29) spe-11(+)*=28, *spsp-1(ude29) spe-11(tn2059*Δ*)*=14, *spe-11(tn2063) spsp-1(+)*=19, *spsp-1(tn2063) spsp-1(ude51*Δ*)*=12. Number of nuclei quantified in (C) *spe-11(tn2063) spsp-1(+)*=55, *spsp-1(tn2063) spsp-1(ude51)*=50. Bars represent mean±s.d. Welch's *t*-test, ****P<0.0001.

As SPSP-1 and SPE-11 colocalize in spermatogenic germlines, we asked if they are reciprocally required for correct localization. To this end we compared the expression and localization patterns of both SPSP-1 and SPE-11 when the other gene was deleted. In the absence of SPE-11, SPSP-1 does not appear as discrete puncta in late prophase nuclei. SPSP-1 instead appears as a diffuse cytoplasmic haze and was largely absent from post-meiotic spermatids (Fig. 6A).

Next, to determine if SPSP-1 is required for proper SPE-11 localization we used CRISPR/Cas9 genome editing to create a precise coding region deletion of *spsp-1* in an mKate2::SPE-11 strain. As this *spsp-1* deletion allele [*spsp-1(ude50)*] differed slightly from both the *spsp-1(syb2478)* and *spsp-1(syb2479)* alleles, we analyzed this allele for fertility and embryonic lethality at 24°C. *spsp-1(ude50)* behaved similarly to *spsp-1(syb2478)* and *spsp-1(syb2479)* (Fig. S4). In the absence of SPSP-1, SPE-11 puncta were observed in late meiotic prophase; however, there were overall fewer puncta per nucleus than in control germ lines (Fig. 6B,C). In post-meiotic spermatids, SPE-11 foci fluorescence was reduced (Fig. 6B). These data indicate that SPSP-1 and SPE-11 are mutually required for localization in spermatogenesis.

## DISCUSSION

This work characterizes a novel sperm gene, *spsp-1*, and its interaction with the paternal-effect gene, *spe-11*. Through a yeast two-hybrid screen, we identified SPSP-1 as an interactor of SPE-11. Furthermore, we demonstrate that SPSP-1 colocalizes with SPE-11 as puncta around late prophase nuclei and spermatid DNA in spermatogenesis. We demonstrate that a loss of *spsp-1* decreases hermaphrodite self-fertility and embryonic development at elevated temperatures. The decrease in hermaphrodite self-fertility is not due to defects in sperm activation and *spsp-1(*Δ*)* oocytes are competent for fertilization. In addition, we show that *spsp-1(*Δ*)* male sperm are capable of activation and fertilization. Interestingly, we found that *spsp-1(*Δ*)* hermaphrodites produce fewer sperm than wild-type hermaphrodites and the absence of SPSP-1 is the likely source of hermaphrodite subfertility.

SPSP-1 is found in both hermaphrodite and male spermatogenic germ lines (Fig. 4), so why do we not observe a defect in male fertility in the *spsp-1* mutants? Unlike hermaphrodites, which produce a finite number of sperm during the last larval stage (L4), male *C. elegans* continually produce sperm throughout their entire life (Singson, 2001; Ward and Carrel, 1979). Thus, males make excess sperm compared to hermaphrodites and a slight decrease in sperm production, as found in *spsp-1* mutant hermaphrodites, could be missed.

*spsp-1* is a 933 bp gene with five exons and four introns (Fig. 1A). No homologs are predicted to exist outside of *Caenorhabditis* nematodes. SPSP-1 protein has a predicted protein length of 310 amino acids and a molecular weight of 34.2 kD (Sternberg et al., 2024). SPSP-1 is predicted to be a largely intrinsically disordered protein (IDP). SPE-11 is also an IDP that has recently been found to form a complex with the oocyte IDP, OOPS-1, at fertilization (Tsukamoto et al., 2025). In addition, AlphaFold 3 predicted these two proteins form structurally ordered regions upon interaction, a phenomenon that has been found for many IDPs (Trivedi and Nagarajaram, 2022; Tsukamoto et al., 2025). Perhaps SPSP-1 and SPE-11 undergo a similar disorder-to-order transition upon interaction in late prophase of spermatogenesis.

Even though SPSP-1 and SPE-11 were found to interact in a yeast two-hybrid screen and to colocalize during spermatogenesis, loss of *spsp-1* exhibits a very mild phenotype compared to the loss of *spe-11*. Loss-of-function mutations of *spe-11* result in severe egg activation defects that include defects in cytokinesis, polar body formation, the block to polyspermy, and synthesis of the eggshell leading to the production of inviable embryos (Hill et al., 1989; Johnston et al., 2010; L'Hernault et al., 1988; McNally and McNally, 2005; Sato and Sato, 2011; Tsukamoto et al., 2025). *spsp-1* mutant hermaphrodites exhibit a reduction in brood size (Fig. 1C,D). However, unlike *spe-11* mutants, *spsp-1* mutants are still capable of producing embryos with ∼80% of the embryos produced resulting in viable progeny (Fig. S1). Furthermore, unlike *spe-11* mutants *spsp-1(*Δ*)* embryos have intact, impermeable eggshells with most embryos arresting prior to elongation (Figs S1D, S5). *spe-11* mutant embryos make defective eggshells, fail to complete oocyte meiosis and to produce polar bodies (Hill et al., 1989; Johnston et al., 2010; L'Hernault et al., 1988; McNally and McNally, 2005; Sato and Sato, 2011). SPSP-1 and SPE-11 colocalize during late prophase in spermatogenesis and in post-meiotic sperm and are reciprocally required for proper localization (Fig. 6). Even though there are lower levels of SPE-11 and the remaining amounts are mislocalized in *spsp-1(*Δ*)* mutant germ lines, there appears to be sufficient amounts of SPE-11 in the mutant sperm for egg activation functions. Taken together, SPSP-1 does not appear to be involved in egg activation and may have functions independent of SPE-11.

Although SPSP-1 was discovered through an interaction with SPE-11, we found that loss of *spsp-1* has distinct phenotypes from those of *spe-11*. First, *spsp-1(*Δ*)* causes a reduction in hermaphrodite self-sperm. Lower sperm numbers can be the result of decreased cell proliferation in the stem cell niche of the germ line (proliferative zone). However, neither SPSP-1 nor SPE-11 are present in the proliferative zone, suggesting that these proteins are unlikely to play a role in mitotic proliferation. Nevertheless, it remains possible that SPSP-1 and SPE-11 are present at levels below the detection threshold of our assays. Another possible cause of fewer sperm in hermaphrodites could stem from defects in the spermatogenesis to oogenesis switch. The hermaphrodite germ line initially produces sperm, creating approximately 150 sperm per gonad arm, then at the L4 to adult molt the germ line permanently switches to oogenesis (Hubbard and Greenstein, 2005). If *spsp-1(*Δ*)* germ lines spend less time in spermatogenesis before switching to oogenesis, fewer sperm would be produced. A third possibility is that defects in chromosome segregation in spermatogenesis could produce sperm lacking DNA. As we quantified sperm through DAPI staining, sperm lacking DNA would not be captured in the quantification. However, we believe this is the least likely possibility as *C. elegans* sperm that lack DNA are still capable of fertilization and produce wild-type broods, albeit dead embryos are produced (Sadler and Shakes, 2000). In addition to fewer sperm, *spsp-1(*Δ*)* have a small but significant embryonic lethal phenotype (Fig. S1). We also found that embryonic lethality is rescued in the reciprocal mating crosses (Fig. S2). The source of the embryonic lethality is unknown, and SPSP-1 was not observed in embryos. Perhaps embryonic lethality is the result of some yet unknown function with SPE-11 after fertilization. Another possibility is that the embryonic lethality is due to *spsp-1* sperm defects that are not captured in these assays. Male-derived sperm are deposited into the uterus and must travel to the spermatheca, the site of fertilization (L'Hernault, 2006). Although we did not observe defects in sperm morphology or sperm activation (Fig. 3C), if a small proportion of the male sperm have defects, the sperm without defects would be more competitive, reach the spermatheca and available for fertilization. Future experiments will be important to decipher the multiple roles of the novel SPSP-1 protein.

## MATERIALS AND METHODS

### Strains

Standard culturing conditions were used to maintain the *C. elegans* strains used in this study (Table S1). All worms were maintained on Modified Youngren's, Only Bacto-peptone (MYOB) plates seeded with *E. coli* OP50.

### CRISPR/Cas9

The *spsp-1(syb2478Δ)* and *spsp-1(syb2479Δ)* alleles were created using CRISPR/Cas9 genome editing by SunyBiotech Corporation (Fu Jian Province, China). The *spsp-1(ude50Δ)* and *spsp-1(ude51Δ)* alleles were generated using CRISPR/Cas9 genome editing via the clone-free method (Paix et al., 2015). The gonads of adult hermaphrodites were injected with a mix using the following components: Cas9 protein (2.5 μg, IDT), *dpy-10* crRNA (25 μM, IDT), *dpy-10(cn64)* repair oligonucleotide (22 μM), universal tracrRNA (60 μM, IDT), an allele-specific crRNA (50 μM) and an allele-specific repair oligonucleotide (28 μM). The crRNA for the 5′ end of the gene was 5′- caatagacaacaaccatcgATGG −3′ and the crRNA for the 3′ end of the gene was 5′- cctgattgattttctagagaata −3′ with the PAM sites underlined and the exons capitalized.

The repair oligo to create a *spsp-1* ORF deletion of specific sequences was 5′-ctcgaatatttcgattcaatagacaacaaccatcgtgaTGAttgatctctgtaatgatatcacctgattgattttctagagaata aaatgatgatttgaa-3′ with a stop codon marked with capital letters.

The *spsp-1(ude29)* and *spsp-1(ude64)* endogenous fluorescent reporter tag insertions were generated as described in Vicencio et al. (2019). The gonads of adult hermaphrodites were injected with a mix using the following components: Cas9 protein (2.5 μg, IDT), *dpy-10* crRNA (25 μM, IDT), *dpy-10(cn64)* repair oligonucleotide (22 μM), universal tracrRNA (60 μM, IDT), an allele-specific crRNA (50 μM) and an allele-specific repair oligonucleotide (28 μM). The crRNA for EGFP::SPSP-1 step 1 insertion was 5′-caatagacaacaaccatcgATGG-3′ and the repair oligo for EGFP::SPSP-1 step 1 was 5′-gaatatttcgattcaatagacaacaaccatcgatgtccaagggagaggagctcttcaccggagtcgtcccaatcctcgtcgagctcgacggagtcaaggagttcgtcaccgctgccggaatcacccacggaatggacgagctctacaaggccaagtctaactcgaacaatagccacaatgaggtaag-3′. Once step 1 was completed, the protocol follows the instructions in Vicencio et. al., and the insertion of the EGFP complete fragment was conducted using the universal crRNA for EGFP, which was 5′- cgucgagcucgacggaguca-3′.

### Brood size assay

Brood sizing was conducted as in Kwah and Jaramillo-Lambert (2023). L4 hermaphrodites of the indicated genotypes were singled out onto individual 35 mm MYOB plates and incubated at either 20°C, 24°C, or 25°C. Following a 24 h incubation period to lay self-progeny, each hermaphrodite was transferred onto a fresh plate. This was repeated for three consecutive days. 24 h after the adult worms were removed each plate was observed and the total larvae and embryos were counted. Mating assays followed the same strategy as above, except a one male to one hermaphrodite/female ratio was used for mating. L4 males and hermaphrodites/females were transferred onto the same plates simultaneously for three consecutive days. Data in graphs represent three biological replicates with at least ten hermaphrodites for each replicate.

### Oocyte quantification

Oocyte quantification was done following the same strategy for brood sizing with the following modifications. Laid oocytes were counted 24 h after each adult transfer to distinguish the oocytes from a dead embryo.

### Whole-mount DAPI staining

For sperm quantification assays, five to ten animals were picked onto a coverslip containing 5 μl M9 buffer. For imaging of *spsp-1(*Δ*)* dead embryos, the dead embryos were picked from agar plates and placed onto a coverslip with 5 μl M9 buffer. The M9 buffer was then wicked away with a Kimwipe and 15 μl of room temperature 100% methanol was added to the coverslip. Once the methanol evaporated 12 μl of 2 μg/ml DAPI diluted in sterile water was immediately added. A slide was placed face down on the coverslip, sealed with clear nail polish, and incubated in the dark for 30 min before imaging. The slides were imaged on a Zeiss LSM980 confocal microscope (Carl Zeiss, Inc., Gottingen, Germany) using a 40× objective with Z-stack intervals of 0.21 μm.

### Sperm quantification

Z-stack images of whole worms were obtained using a Zeiss LSM980 confocal microscope (Carl Zeiss, Inc., Gottingen, Germany) using a 63× objective and Zen software. For each spermatheca imaged, a full range of focal planes were selected to include the spermatheca, with a constant Z-step of 0.2 μm. Images were taken using identical imaging parameters, but brightness and contrast were adjusted to allow for better visualization. Images used for sperm quantification were visualized in 3D using Imaris x64 10.2.0 software and nuclei were recognized and filtered using volume, sphericity, and voxel quantity in the surfaces function.

### Sperm activation assays

L4 males were isolated from hermaphrodites for 24 h in a 24°C incubator. For inactivated male sperm, males were dissected on a charged slide in SM buffer (50 mM HEPES, 45 mM NaCl, 25 mM KCl, 1 mM MgSO_4_, 5 mM CaCl_2_, 10 mM Dextrose, pH 7.8). The dissected samples were covered with a 22×22 mm glass coverslip and incubated in a humid chamber for 15 min before sealing the coverslip to the slide with clear nail polish. After the incubation, sperm were immediately imaged with a 40× objective lens with DIC optics on a Zeiss AxioObserver (Carl Zeiss Inc. Gottingen, Germany). *In vitro* activated male sperm were isolated and dissected the same as the inactivated male sperm with the addition of 20 μg/ml Pronase (Sigma, P1547) in the SM buffer. For *in vivo* activated hermaphrodite sperm, L4 hermaphrodites were isolated from males, incubated at 24°C for 24 h, and dissected in SM buffer without Pronase.

### Live imaging of SPSP-1 and SPE-11 in hermaphrodite and male spermatogenesis

L4 hermaphrodites and adult males were staged by placing them onto fresh MYOB plates for 24 h prior to imaging. For imaging of live worms, a slide was prepared consisting of a 30 μl 2% agarose gel pad that was flattened between two slides and allowed to solidify. Worms were mounted on the agarose pad with a droplet of 20 mM levamisole and allowed to paralyze. A coverslip was placed over the agarose pad and the slides imaged with an Andor Dragonfly spinning disk confocal microscope (Oxford Instruments) using a Plan Apo 63× objective and a Zyla sCMOS camera (Oxford Instruments) with a Z-stack interval of 0.2 μm. Images were analyzed using Imaris Microscope Image Analysis software (Oxford Instruments) and ImageJ (Fiji) (Schindelin et al., 2012). All images were obtained using identical parameters, with brightness and contrast adjusted for better visualization.

### Immunostaining

Male germline dissection, immunofluorescence, and DAPI staining (Fig. 6) was performed as in (Rourke and Jaramillo-Lambert, 2022). Incubations at the non-permissive temperature of 24°C were done for 16-24 h. Gonads were dissected in 30 μl of Egg buffer and 0.1% Tween 20 on a glass coverslip followed by removal of 15 μl of the buffer. The removed buffer was replaced with 2% paraformaldehyde. 15 μl was removed, and a slide was placed on top of the coverslip. The samples were allowed to fix for 5 min. After fixation, the slides were immersed in liquid nitrogen. After freezing, the coverslips were quickly removed, and the slides were placed in −20°C 100% methanol for 1 min. The slides were then washed once in 1× PBS+0.5% Triton X-100 for 10 min followed by two washes in PBST (1× PBS+0.1% Tween 20) for 5 min each. The slides were blocked in 0.7% BSA in PBST for 1 h. After blocking, 50 μl of primary antibody [rabbit anti-GFP (1:500, Novus Biologicals, NB600-308) or rabbit anti-RFP (1:100, Invitrogen, PA5-34974)] was added. A parafilm coverslip was placed on top of the samples and the slides were incubated overnight in a dark, humid chamber. After the primary antibody incubation, the slides were washed three times for 5 min each in PBST. 50 μl of secondary antibody [goat anti-rabbit Alexa Fluor-568 (1:200, Invitrogen, A11036) or goat anti-rabbit Alexa Fluor-488 (1:200, Invitrogen, A11034)] was added to the slides. The slides were incubated in a dark humid chamber for 2 h. The slides were then washed three times and counterstained with DAPI (2 μg/ml) for 5 min, then washed once in PBST for 5 min. A glass coverslip was placed on top of the samples with Vectashield (Invitrogen, P36980). Z-stack images of post-meiotic male sperm were obtained using a Zeiss LSM980 confocal microscope (Carl Zeiss, Inc. Gottingen, Germany) using a 40× objective lens, with 0.2 μm Z-steps. Images are Z-projections through the full Z-stack range. Image processing and analysis was performed using Fiji (Schindelin et al., 2012).

### Eggshell permeability assay

L4 hermaphrodites of the indicated genotypes were grown on a 35 mm MYOB plate for either 24 h or 48 h at 24°C. A 40 μM FM4-64 lipophilic dye solution was made in 1X egg buffer (118 mM NaCl, 48 mM KCl, 2 mM CaCl_2_ ⋅ 2H_2_O, 2 mM MgCl_2_ ⋅ 6H2O, 25 mM HEPES, pH 7.4). After the incubation period at 24°C, the worms were picked onto a coverslip with 5 μl of the 40 μM FM4-64 dye solution and dissected at the vulva to release the embryos. The coverslip was then placed onto a slide with a 2% agarose pad. A thin line of petroleum jelly was placed around the agarose pad prior to placing the coverslip on top to avoid crushing the embryos. The slide was immediately imaged with a 40× objective lens on a Zeiss Axio Observer (Carl Zeiss Inc. Gottingen, Germany). Images were analyzed using ZEN software.

### Statistical analysis

Statistical analyses were performed using GraphPad Prism 6. All experiments were conducted with a minimum of three biological replicates. Error bars in embryonic viability graphs indicate standard error of the mean (s.e.m.). Error bars in all other graphs indicate standard deviation. The statistical tests used for each experiment are indicated in the figure legends. Statistical tests, *P*-values, and samples numbers for each figure are listed in Table S2.

## Supplementary Material



10.1242/biolopen.062634_sup1 Supplementary information
