# Supplementary material for: SPSP-1 is a sperm protein required for C. elegans hermaphrodite sperm production and embryogenesis
Source: Biol Open. 2026 Jun 30;15(6):bio062634. doi: 10.1242/bio.062634 (PMC13382835; doi:10.1242/bio.062634)
Supplement: Supplementary information [file biolopen-15-062634-s1.pdf]

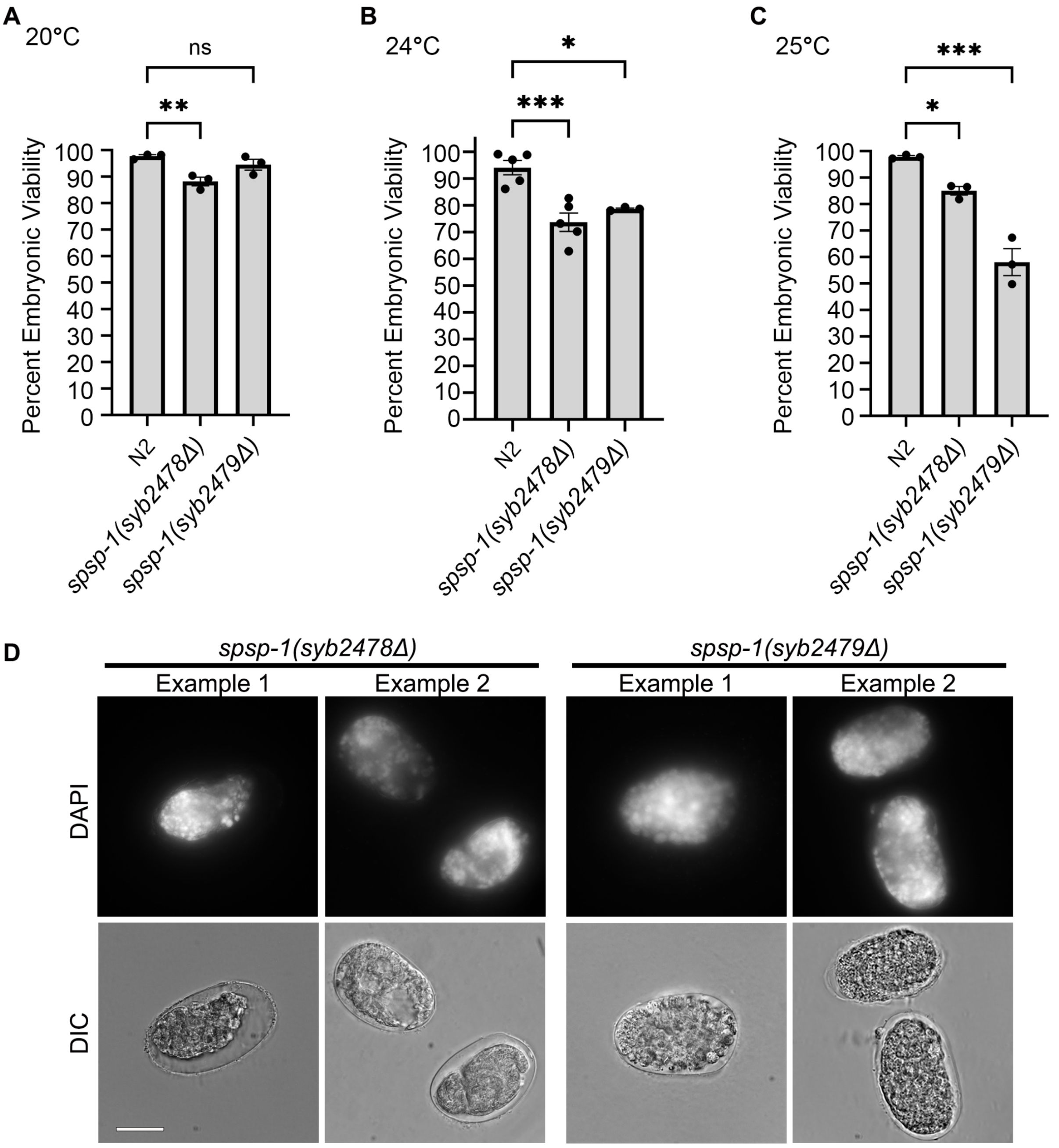

Fig. S1. ***spsp-1*** **mutants result in decreased embryonic viability.** Average percent embryonic viability from N2, *spsp-1(syb2478Δ),* and *spsp-1(syb2479Δ)* at (A) 20°C, (B) 24°C, and (C) 25°C. Sample sizes (number of individual hermaphrodite broods scored) at 20°C: N2 N=21, *spsp-1 (syb2478Δ)* N=30, and *spsp-1(syb2479Δ)* N=24; 24°C N2 N=28, *spsp-1(syb2478Δ)* N=35, and *spsp-1(syb2479Δ)* N=30; 25°C N2 N=25, *spsp-1 (syb2478Δ)* N=22, and *spsp-1 (syb2479Δ)* N=26. Statistics were performed using a one-way ANOVA. ns=not significant, *p<0.05, **p=0.008, ***p≤0.0008. (D) Examples of DAPI stained dead embryos from *spsp-1(syb2478Δ)* and *spsp-1(syb2479Δ)* at 24°C. Scale bar= 20 µm.

Biology Open • Supplementary information

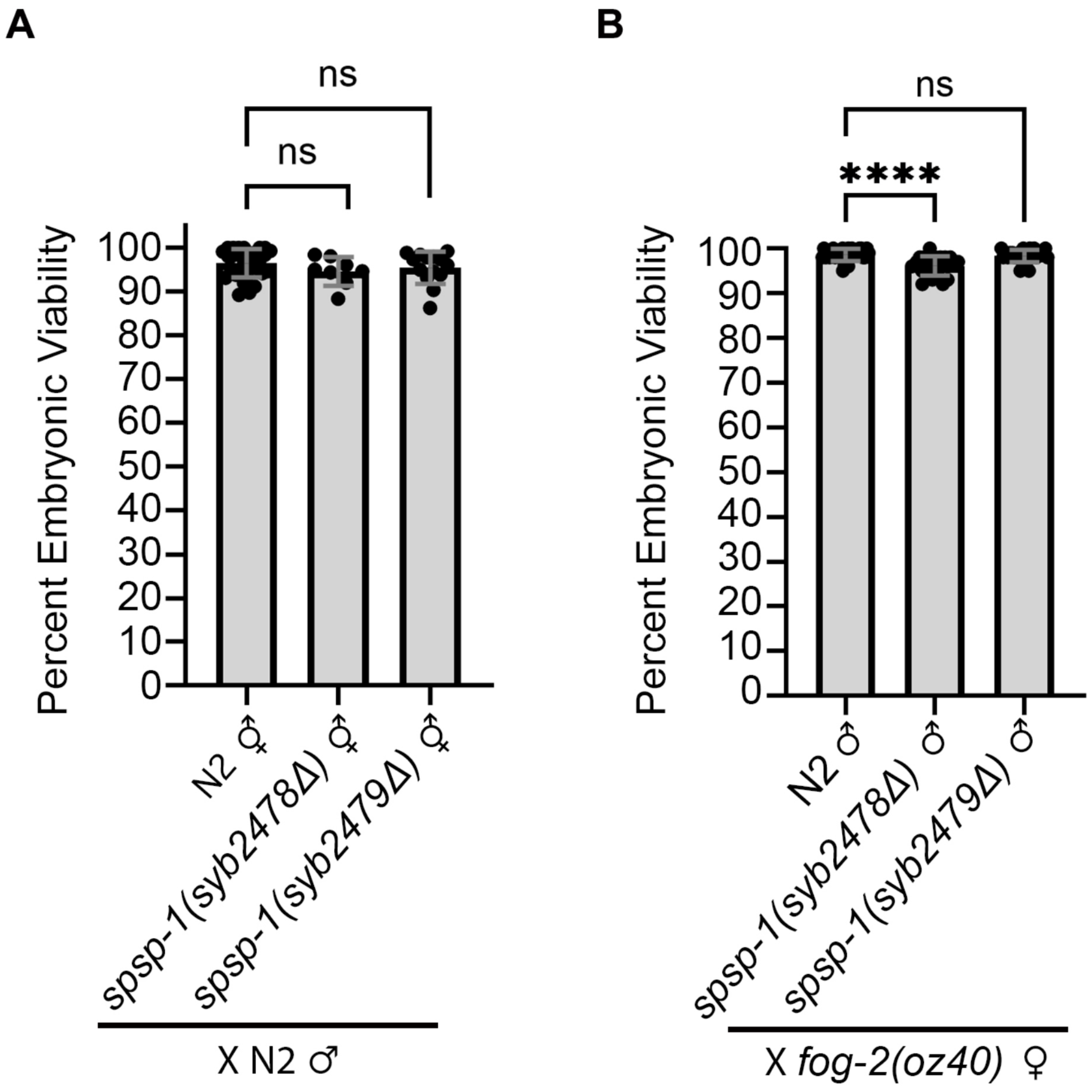

Fig. S2. Embryonic viability from reciprocal mating crosses.

(A) Average percent embryonic viability from N2♂ crossed with N2, *spsp-1 (syb2478),* and *spsp-1 (syb2479)* ♀ at 24°C. For these experiments a single male was mated to a single hermaphrodite for 24 h and the resulting progeny scored for three days. Sample sizes (number of individual crosses scored): N2 N=31, *spsp-1 (syb2478Δ)* N=8, and *spsp-1 (syb2479Δ)* N=14. (B) Average percent embryonic viability from N2, *spsp-1(syb2478),* and *spsp-1(syb2479)* ♂ crossed with *fog-2(oz40)* ♀ at 24°C. For these experiments a single male was mated to a single female for 24 h and the resulting progeny scored for three days. Sample sizes (number of individual crosses scored): N2 N=30, *spsp-1(syb2478Δ)* N=23, and *spsp-1(syb2479Δ)* N=27. Statistics were performed using a one-way ANOVA. ns=not significant, ****p≤0.0001.

Biology Open • Supplementary information

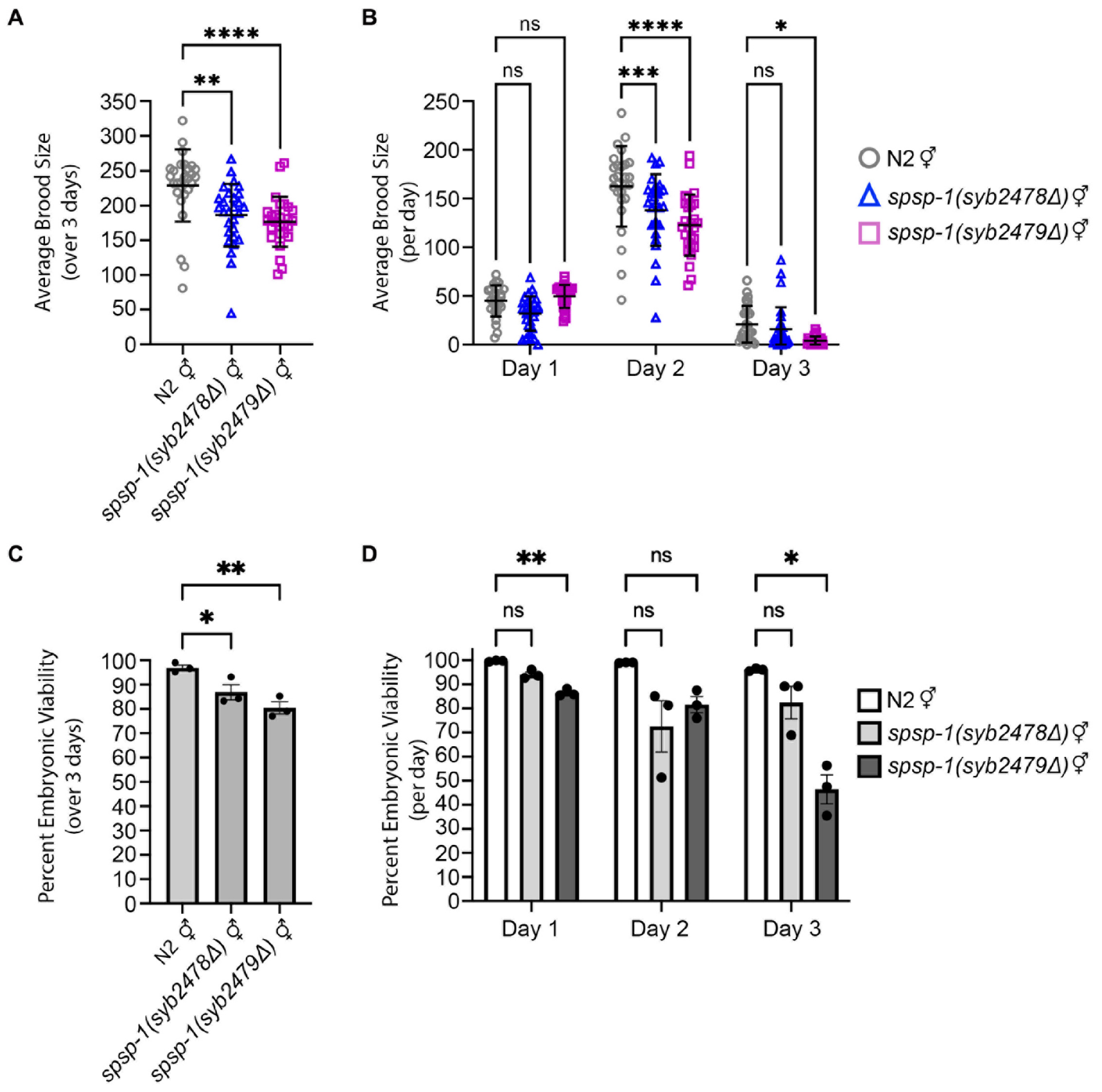

Fig. S3. Brood size and embryonic viability of *spsp-1* mutants. Brood size and embryonic viability data presented here was collected at the same time and from the same plates as the oocyte data in Fig. 3. (A) Average brood size from N2, *spsp-1(syb2478),* and *spsp-1(syb2479)* at 24°C presented as an average of the 72-h assay window. (B) Average percent brood sise from N2, *spsp-1(syb2478),* and *spsp-1(syb2479)* at 24°C presented by day. (C) Embryonic viability of N2, *spsp-1(syb2478),* and *spsp-1(syb2479)* at 24°C presented as an average of the 72-h assay window. (D) Average percent embryonic viability from N2, *spsp-1 (syb2478),* and *spsp-1 (syb2479)* at 24°C presented by day. Each graph is the data from three replicates. Total number of hermaphrodite broods examined: N2 N=27, *spsp-1(syb2478Δ)* N=28, and *spsp-1(syb2479Δ)* N=27. (A & C) One-way ANOVA; *p<0.05, **p=0.005. (B & D) Two-way ANOVA; ns=not significant, *p<0.05, **p=0.006.

Biology Open • Supplementary information

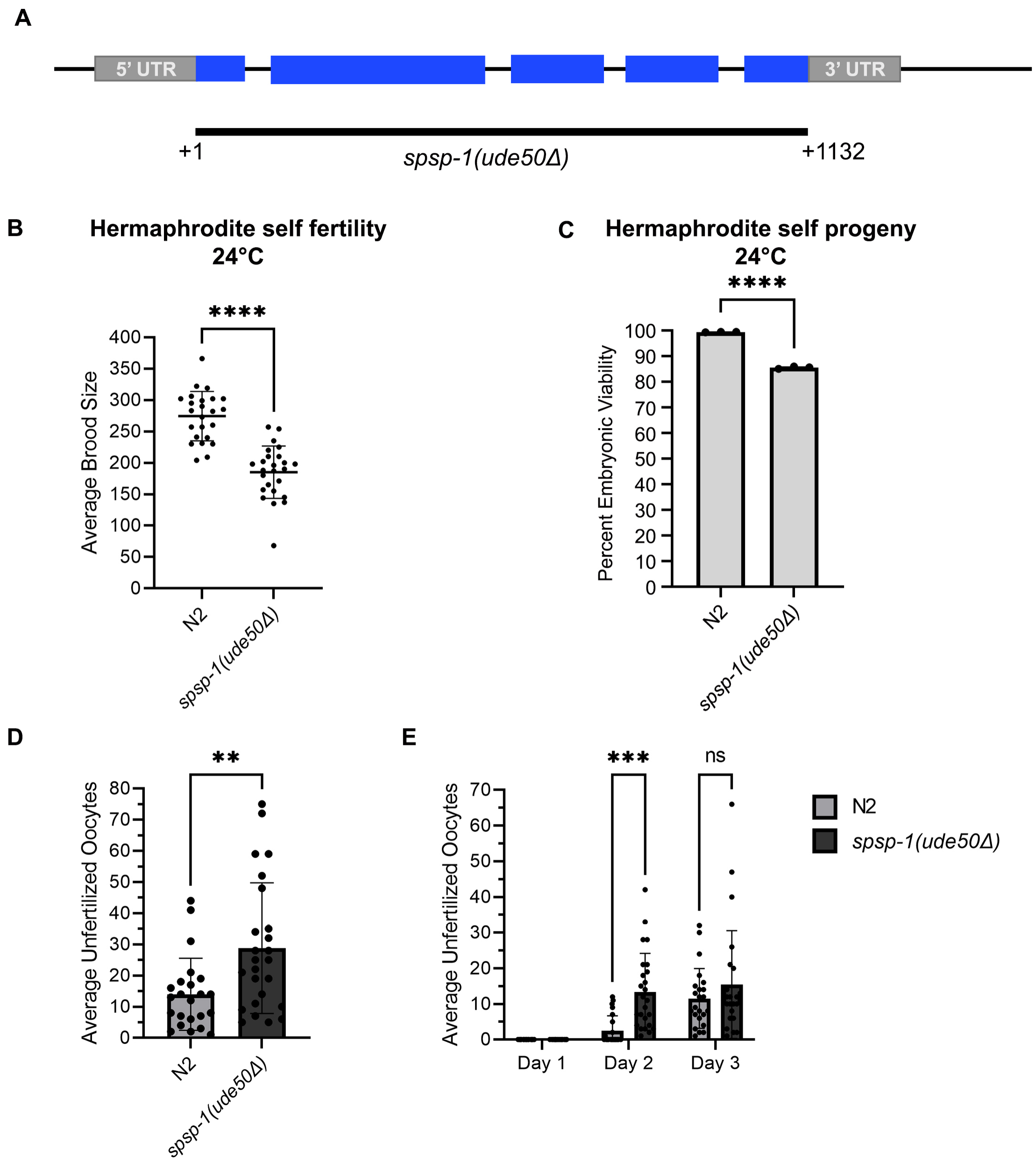

Fig. S4. Characterization of *spsp-1 (ude50Δ).* (A) *spsp-1 (ude50Δ)* is a precise 1132 bp deletion of the entire coding sequence. (B) Average brood sizes of N2 and *spsp-1 (ude50Δ)* hermaphrodites at 24°C. (C) Average percent embryonic lethality at 24°C. N2 N=24 and *spsp-1 (ude50Δ)* N=26. Statistics were performed using a Welch's T-test. ***p≤0.0005, ****p<0.0001. (D) The number of unfertilized oocytes of N2 and *spsp-1 (ude50Δ)* hermaphrodites at 24°C presented as an average of the 72-h assay window. (E) The average number of oocytes of N2 and *spsp-1(ude50Δ)* mutant hermaphrodites at 24°C by day. Sample sizes: N2 N=23 and *spsp-1(ude50Δ)* N=25. Statistics were performed using one-way ANOVA. ns=not significant, ***p≤0.0005.

Biology Open • Supplementary information

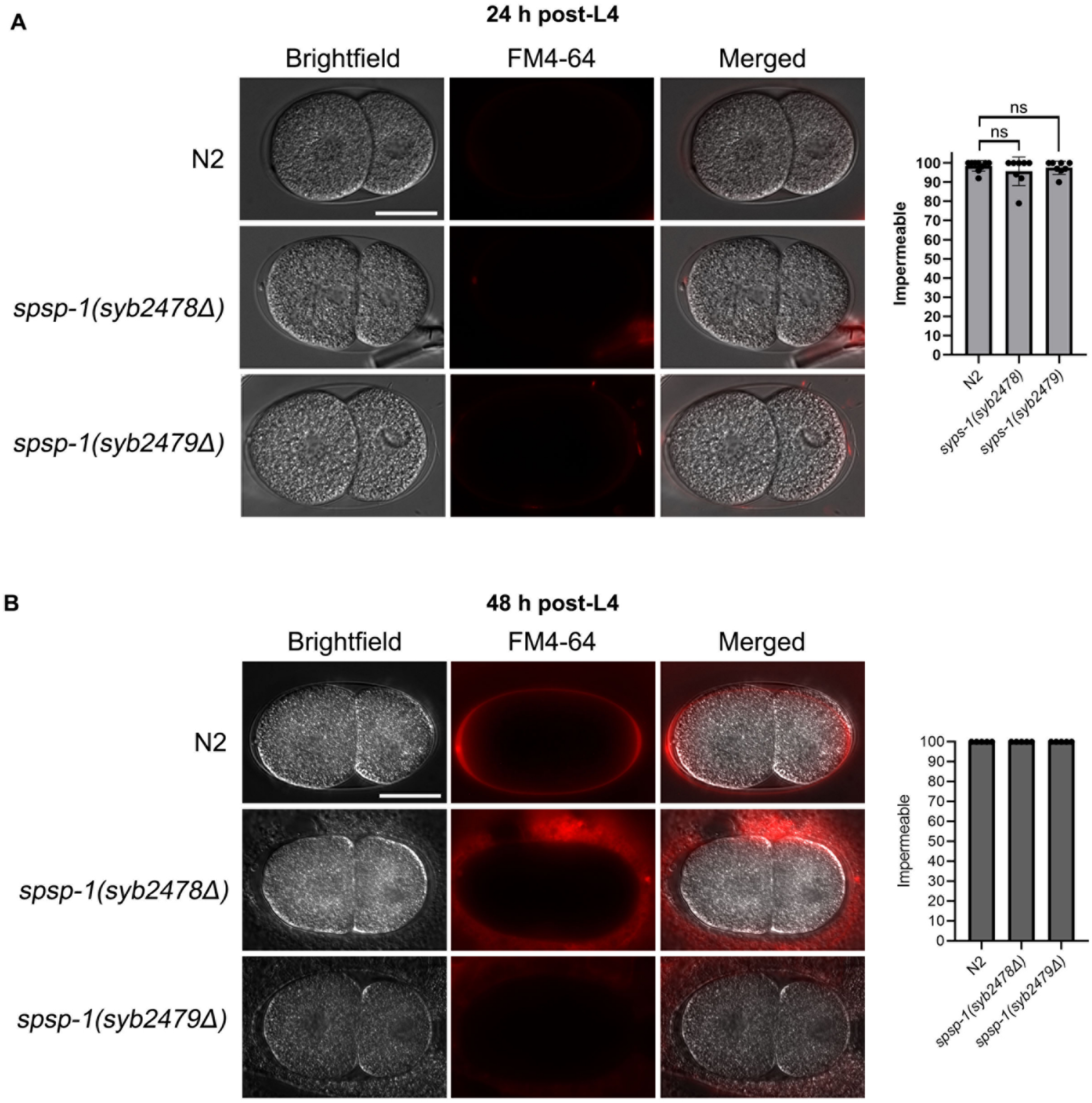

Fig. S5. *spsp-1(Δ)* mutant eggshells are impermeable. FM4-64 staining of C. *elegans* embryo eggshells 24 h post L4 staging (A) and 48 h post L4 staging (B) at 24°C. Sample sizes: 24h N2= 239, *spsp-1 (syb2478Δ)*= 156, *spsp-1(syb2479Δ)*= 242. 48 h N2= 114, *spsp-1(syb2478Δ)*= 71, *spsp-1 (syb2479Δ)*= 77. Chi-square test ns= not significant. Standard statistical comparisons for (B) could not be calculated because all samples had an SD=0.

Table S1. Strains used in this study

The following table includes the general maintenance temperature, strain number, and corresponding genotypes. General maintenance was performed at 20°C on 60 mm MYOB agarose plates inoculated with *E. coli* OP50.

Strain	Genotype/Chromosome location	Citation/Source
N2	Bristol Wild-type	CGC
PHX2478	*spsp-1(syb2478Δ)* I	This study
PHX2479	*spsp-1(syb2479Δ)* I	This study
AJL64	*spsp-1(ude29)* [*egfp::spsp-1*] I; *itIs37* [pAA64: *pie-1p::mCherry::his-58* + *unc-119(+)*]	This study
AJL70	*spsp-1(ude29)* [*egfp::spsp-1*] *spe-11(tn2063* [*mKate2::tev::3xflag::spe-11*]*)* I	This study
DG5281	*mkate2::spe-11(tn2063[mKate2::tev::3xflag::spe-11])* I	Tsukamoto, Kwah, et. al. (2025)
DG4915	*his-72 (uge30[gfp::his-72])* III; *fog-2(oz40)* V	David Greenstein UMN
AJL112	*mkate2::spe-11(tn2063)* [*mKate2::tev::3xflag::spe-11*] *spsp-1(ude51Δ)* I	This study
AJL125	*mkate2::spe-11(tn2063)* [*mKate2::tev::3xflag::spe-11*] *spsp-1(ude51Δ) I; him-8(e1489)* IV	This study
AJL111	*spsp-1(ude50Δ)* I	This study
AJL170	*spsp-1(ude29)* [*egfp::spsp-1*] *spe-11(tn2059Δ)/tmc18 I; him-8(e1489) IV*	This study

Biology Open • Supplementary information

Table S2. N numbers, replicates, and statistical tests

Figure 1

	genotype	Total Number of Broods over 3 reps	avg brood	S.D.	Welch's T-test for brood sizes		
1B	N2	21	242.4	24.1	**20°C**		
	spsp-1(syb2478)	30	225.6	40.9	N2 vs. spsp-1(syb2578)	p=0.0718	n.s.
	spsp-1(syb2479)	24	232.1	48.5	N2 vs. spsp-1(syb2579)	p=0.3644	n.s.
1C	N2	28	202.0	87.6	**24°C**		
	spsp-1(syb2478)	35	125.1	50.4	N2 vs. spsp-1(syb2578)	p=0.0002	***
	spsp-1(syb2479)	30	134.1	31.9	N2 vs. spsp-1(syb2579)	p=0.0005	***
1D	N2	25	97.9	1.5	**25°C**		
	spsp-1(syb2478)	22	84.6	7.1	N2 vs. spsp-1(syb2578)	p<0.0001	****
	spsp-1(syb2479)	26	56.9	23.1	N2 vs. spsp-1(syb2579)	p<0.0001	****

Biology Open • Supplementary information

Figure 2

Figure panel	genotype	Total Number of Broods over 3 reps	avg brood	S.D.	one-way ANOVA	p-value	
2A	N2 ⚥ X N2 ♂	20	247.1	146.1	**2A**		
	spsp-1(syb2478) ⚥ X N2 ♂	19	247.2	118.3	N2 ⚥ X N2 ♂ vs. spsp-1(syb2578) ⚥ X N2 ♂	>0.9999	n.s.
	spsp-1(syb2479) ⚥ X N2 ♂	27	212.0	115.4	N2 ⚥ X N2 ♂ vs. spsp-1(syb2579) ⚥ X N2 ♂	0.5407	n.s.
2B	N2 ♂ X *fog-2(oz40)* ♀	30	291.1	135.0	**2B**		
	spsp-1(syb2478) ♂ X *fog-2(oz40)* ♀	30	288.6	129.0	N2 ♂ X fog-2(oz40) ♀ vs. spsp-1(syb2478) ♂ X fog-2(oz40) ♀	0.9957	n.s.
	spsp-1(syb2479) ♂ X *fog-2(oz40)* ♀	30	313.3	121.8	N2 ♂ X fog-2(oz40) ♀ vs. spsp-1(syb2479) ♂ X fog-2(oz40) ♀	0.7286	n.s.
	**genotype**	**Total Number of Hermaphrodites**	**avg sperm quantity**	**S.D.**	**one-way ANOVA**	**p-value**	
2C	N2	30	142.6	26.5	**3A (6 h)**		
	spsp-1(syb2478)	30	89.9	40.9	N2 vs. spsp-1(syb2578)	p<0.0001	****
	spsp-1(syb2479)	30	91.2	36.8	N2 vs. spsp-1(syb2579)	p<0.0001	****
2D	N2	30	67.9	31.6	**3B (24 h)**		
	spsp-1(syb2478)	30	66.6	36.6	N2 vs. spsp-1(syb2578)	p=0.9831	n.s.
	spsp-1(syb2479)	30	57.7	33.2	N2 vs. spsp-1(syb2579)	p=0.4015	n.s.
2E	N2	30	56.0	25.8	**3C (36 h)**		
	spsp-1(syb2478)	30	49.1	24.2	N2 vs. spsp-1(syb2578)	p=0.4408	n.s.
	spsp-1(syb2479)	30	22.7	18.6	N2 vs. spsp-1(syb2579)	p<0.0001	****
2F	N2	30	9.2	9.5	**3C (48 h)**		
	spsp-1(syb2478)	30	6.6	6.6	N2 vs. spsp-1(syb2578)	p=0.4364	n.s.
	spsp-1(syb2479)	30	6.7	10.1	N2 vs. spsp-1(syb2579)	p=0.4634	n.s.

Biology Open • Supplementary information

Figure 3

Figure panel	genotype	Total number of Hermaphrodites	avg oocyte quantity	S.D.
**3A & 3B**	N2	27	20.8	14.4
	spsp-1(syb2478)	28	36.2	16.9
	spsp-1(syb2479)	27	63.0	29.6

one-way ANOVA (Average over 3 days)	p-value	
**3A**		
N2 vs. spsp-1(syb2578)	p=0.0174	*
N2 vs. spsp-1(syb2579)	p<0.0001	****

two-way ANOVA (Average by day)	p-value	
**Day 1**		
N2 vs. spsp-1(syb2578)	p=0.996	n.s.
N2 vs. spsp-1(syb2579)	p>0.9999	n.s.
**Day 2**		
N2 vs. spsp-1(syb2578)	p=0.0380	*
N2 vs. spsp-1(syb2579)	p<0.0001	****
**Day 3**		
N2 vs. spsp-1(syb2578)	p=0.0196	**
N2 vs. spsp-1(syb2579)	p<0.0001	****

		Total number of sperm	
		Inactive	Activated
**3C**	N2	139	128
	spsp-1(syb2478)	125	129
	spsp-1(syb2479)	102	104

		Total number of Hermaphrodites Activated
**3D**	N2	149
	spsp-1(syb2478)	100
	spsp-1(syb2479)	49

Biology Open • Supplementary information

Figure 4

Figure panel	genotype	Total number of Males
**4A & B**	*spsp-1(ude29); itIs37*	29
		**Total number of Hermaphrodites**
**4C & D**	*spsp-1(ude29); itIs37*	29

Figure 5

Figure panel	genotype	Total number of Males
**5A & B**	*spsp-1(ude29); spe-11(tn2063)*	26
		**Total number of Hermaphrodites**
**5C & D**	*spsp-1(ude29); spe-11(tn2063)*	20

Figure 6

Figure panel	genotype	Total number of germ lines	
**6A**	*spsp-1(ude29* [*egfp::spsp-1*]*); him-8(e1489)*	28	
	spe-11(tn2059Δ) spsp-1(ude29 [*egfp::spsp-1*]*)/tmC18; him-8(e1489)*	14	
		**Total number of germ lines**	
**6B**	*spe-11(tn2063[mKate2::tev::3xflag::spe-11]), him-8(e1489)*	19	
	spe-11(tn2063) [*mKate2::tev::3xflag::spe-11*] *spsp-1(ude51Δ); him-8(e1489)*	12	
		**Total number of nuclei**	**Welch's T-test for number of puncta surrounding nuclei**
**6C**	*spe-11(tn2063[mKate2::tev::3xflag::spe-11]), him-8(e1489)*	55	
	spe-11(tn2063) [*mKate2::tev::3xflag::spe-11*] *spsp-1(ude51Δ); him-8(e1489)*	50	*spe-11(tn2063) spsp-1(+) vs. spe-11(tn2063) spsp-1(ude51)* p<0.0001 ****

Supplemental Figure 1

	genotype	Total Number of Broods over 3 reps	avg emb	S.E.M.	One way ANOVA for embryonic viability		
S1A	N2	21	97.7	0.58	**20°C**		
	spsp-1(syb2478)	30	88.2	1.63	N2 vs. spsp-1(syb2578)	p=0082	**
	spsp-1(syb2479)	24	94.5	2.00	N2 vs. spsp-1(syb2579)	p=0.3115	ns
S1B	N2	28	94.0	2.68	**24°C**		
	spsp-1(syb2478)	35	73.7	3.48	N2 vs. spsp-1(syb2578)	p=0.0008	***
	spsp-1(syb2479)	30	78.6	0.38	N2 vs. spsp-1(syb2579)	p=0.0128	*
S1C	N2	25	97.9	0.41	**25°C**		
	spsp-1(syb2478)	22	85.1	1.60	N2 vs. spsp-1(syb2578)	p=0.0459	*
	spsp-1(syb2479)	26	58.1	5.10	N2 vs. spsp-1(syb2579)	p=0.0002	***

	genotype	Total Number of Embryos Scored over 2 reps
S1D	N2	N/A
	spsp-1(syb2478)	8
	spsp-1(syb2479)	9

Biology Open • Supplementary information

Supplemental Figure 2

Figure panel	genotype	Total Number of Broods over 3 reps	avg emb	S.E.M.	one-way ANOVA	p-value	
S2A	N2 ⚥ X N2 ♂	31	96.5	0.6	**S2A**		
	spsp-1(syb2478) ⚥ X N2 ♂	8	94.6	1.2	N2 ⚥ X N2 ♂ vs. spsp-1(syb2578) ⚥ X N2 ♂	p=0.2833	n.s.
	spsp-1(syb2479) ⚥ X N2 ♂	14	95.4	1.0	N2 ⚥ X N2 ♂ vs. spsp-1(syb2579) ⚥ X N2 ♂	p=0.5227	n.s.
S2B	N2 ♂ X *fog-2(oz40)* ♀	30	98.6	0.2	**S2B**		
	spsp-1(syb2478) ♂ X *fog-2(oz40)* ♀	23	96.1	0.5	N2 ♂ X fog-2(oz40) ♀ vs. spsp-1(syb2478) ♂ X fog-2(oz40) ♀	p<0.0001	****
	spsp-1(syb2479) ♂ X *fog-2(oz40)* ♀	27	98.3	0.3	N2 ♂ X fog-2(oz40) ♀ vs. spsp-1(syb2479) ♂ X fog-2(oz40) ♀	p=0.7706	n.s.

Biology Open • Supplementary information

Biology Open (2026): doi:10.1242/bio.062634: Supplementary information

Supplemental Figure 3

	genotype	Total Number of Broods (3 reps)	avg brood	S.E.M.	One way ANOVA for brood sizes		
SF3A&B	N2	27	228.8	10.00			
	spsp-1(syb2478)	28	186.1	8.51	N2 vs. spsp-1(syb2578)	p=0.0014	**
	spsp-1(syb2479)	27	176.5	6.90	N2 vs. spsp-1(syb2579)	p<0.0001	****
					**two-way ANOVA for brood sizes (Average by day)**		
					**Day 1**		
					N2 vs. spsp-1(syb2578)	p=0.0990	n.s.
					N2 vs. spsp-1(syb2579)	p=0.7201	n.s.
					**Day 2**		
					N2 vs. spsp-1(syb2578)	p=0.0007	***
					N2 vs. spsp-1(syb2579)	p<0.0001	****
					**Day 3**		
					N2 vs. spsp-1(syb2578)	p=0.6604	n.s.
					N2 vs. spsp-1(syb2579)	p=0.0248	*
SF3C&D	N2	27	97.0	1.08	**One way ANOVA for embryonic viability**		
	spsp-1(syb2478)	28	86.9	3.15	N2 vs. spsp-1(syb2578)	p=0.0446	*
	spsp-1(syb2479)	27	80.4	2.50	N2 vs. spsp-1(syb2579)	p=0.0050	**
					**two-way ANOVA for embryonic viability (Average by day)**		
					**Day 1**		
					N2 vs. spsp-1(syb2578)	p=0.0522	n.s.
					N2 vs. spsp-1(syb2579)	p=0.0055	**
					**Day 2**		
					N2 vs. spsp-1(syb2578)	p=0.2284	n.s.
					N2 vs. spsp-1(syb2579)	p=0.0618	n.s.
					**Day 3**		
					N2 vs. spsp-1(syb2578)	p=0.3087	n.s.
					N2 vs. spsp-1(syb2579)	p=0.0258	*

Supplemental Figure 4

Figure panel	genotype	Total Number of Broods (3 reps)	avg brood	S.D.	Welch's T-test for brood sizes		
SF4B	N2	24	274.4	39.4	**24°C**		
	spsp-1(ude50)	26	184.6	40.9	N2 vs. spsp-1(ude50)	p<0.0001	****
		**Total Number of Broods (3 reps)**	**avg emb**	**S.E.M.**	**Welch's T-test for brood sizes**		
SF4C	N2	24	99.4	0.05	**24°C**		
	spsp-1(ude50)	26	85.5	0.27	N2 vs. spsp-1(ude50)	p=0.0003	***
**Figure panel**	**genotype**	**Total number of Hermaphrodites**	**avg oocyte quantity**	**S.D.**	**Welch's T-test (Over three days)**		
SF4D-E	N2	23	14.0	11.6	N2 vs. spsp-1(ude50)	p=0.0040	**
	spsp-1(ude50)	25	28.8	21.0			
					**Ordinary one-way ANOVA (Average by day)**		
					**Day 1**		
					N2 vs. spsp-1(ude50)	p>0.9999	ns
					**Day 2**		
					N2 vs. spsp-1(ude50)	p=0.0002	***
					**Day 3**		
					N2 vs. spsp-1(ude50)	p=0.3466	ns

Supplemental Figure 5					
**Figure panel**	**genotype**	**Total number of embryos**	**Ordinary one-way ANOVA**	**p-value**	
**SF5A**	N2	116			
	spsp-1(syb2478)	91	N2 vs. *spsp-1(syb2478)*	p=0.4098	n.s.
	spsp-1(syb2479)	89	N2 vs. *spsp-1(syb2479)*	p=0.8954	n.s.
**Figure panel**	**genotype**	**Total number of embryos**	**Not tested because SD for all samples = 0**	**p-value**	
**SF5B**	N2	114			
	spsp-1(syb2478)	71	N2 vs. *spsp-1(syb2478)*	Unable to determine	
	spsp-1(syb2479)	77	N2 vs. *spsp-1(syb2479)*	Unable to determine	

Biology Open • Supplementary information
