# Supplementary material for: SPSP-1 is a sperm protein required for C. elegans hermaphrodite sperm production and embryogenesis
Source: Biol Open. 2026 Jun 30;15(6):bio062634. doi: 10.1242/bio.062634 (PMC13382835; doi:10.1242/bio.062634)
Supplement: Supplementary file 1 [file biolopen-15-062634_review_history.pdf]

SPSP-1 is a sperm protein required for C. elegans hermaphrodite sperm production and embryogenesis

Darline Murat, Margaret Nagorski, Ji Kent Kwah, Shannon Pfeiffer, Nancy Duker, Frank Döring and Aimee Jaramillo-Lambert

10.1242/bio.062634

Editor: Sandhya Koushika

Review timeline
Original submission: 20 April 2026
Editorial decision: 27 April 2026
First revision received: 31 May 2026
Accepted: 4 June 2026

Original submission

First decision letter

MS ID#: bio.062634

MS Title: SPSP-1 is a sperm protein required for C. elegans hermaphrodite sperm production and embryogenesis

Authors: Aimee Jaramillo-Lambert, Darline Murat, Margaret Nagorski, Ji Kent Kwah, Shannon Pfeiffer, Nancy Duker and Frank Döring

I have now reached a decision on the above manuscript.

The reviewer reports are shown at the bottom of this email.

As you will see, the reviewers gave favourable reports, but raised some critical points that will require amendments to your manuscript. I hope that you will be able to carry these out, because we would like to be able to accept your paper.

At this stage, we also ask you to ensure your manuscript complies with our formatting guidelines - please see our manuscript preparation guidelines for details. Provided you are able to fully address the referees' comments, we are positive about publication of your paper (we accept over 95% of revision submissions) and therefore hope you won't mind any extra work involved in reformatting your manuscript at this point.

Please upload both a 'clean' version of your Word file, along with a highlighted version clearly showing where you have made changes in the revised manuscript. Please avoid using 'Track changes' in Word files as these are lost in PDF conversion.

I should be grateful if you would also provide a point-by-point response detailing how you have dealt with the points raised by the reviewers in the 'Response to Reviewers' box. Please attend to all of the reviewers' comments. If you do not agree with any of their criticisms or suggestions please explain clearly why this is so.

Reviewer 1

Comments for the author

© 2026. Published by The Company of Biologists under the terms of the Creative Commons Attribution License (https://creativecommons.org/licenses/by/4.0/).

This study identifies a novel sperm protein, SPSP-1, that is associated with SPE-11 and is required for normal levels of hermaphrodite sperm count and for wild-type embryonic viability. Overall, the findings from this study has significant potential for future studies on the paternal contribution of oogenesis and embryogenesis. However, I request minor revisions in data presentation and recommend textual changes that could improve the clarity and impact of the manuscript.

Specific concerns –

1. The data presented in Figure 1 shows that spsp-1 mutants have a reduced brood size. Given that the sperm count is lower at 6h post L4, but not at 24 h post L4, could the authors include day-wise embryonic viability? Most of the brood size is reported as an average over 3 days. It would also be nice to see day-wise brood size trend.
2. The reciprocal mating experiment is an excellent approach to distinguish between sperm vs oocyte defects. I was surprised to see that reciprocal crosses produce similar brood size. Could the authors also include embryonic viability/lethality in these experiments? Again, day-wise embryonic viability would be a useful analysis. Also, the conclusion at line 129…."However, spsp-1 is required for some aspect of hermaphrodite self-fertility" feels vague. Could the authors clarify this further in the Discussion section and improve the writing of this section in the Results?
3. Could the authors also speculate why the sperm count is lower at 6h post L4 but not at 24h post L4 in the discussion? Can the authors comment on the kinetics of spermatogenesis, cell division, or sperm migration in the Discussion?
4. The hermaphrodite sperm count is found to be less at 6h post L4. Could the authors conduct this analysis on mutant males from the reciprocal cross experiments?
5. In Figure 3C, can the percentage of sperm activation be included? Are all the sperms in the experiment being activated? Is it 100%? Can this be made clear in the figure and figure legend?
6. In Figures 3A and 3B, I believe the Y-axis should be average unfertilized oocytes laid and not total oocytes? Could this be changed in the figures and figure legends?
Also, can the day-wise unfertilized oocytes in Figure 3B be correlated to sperm count AND/OR day-wise embryonic viability? It would be interesting to see if day-wise brood size, sperm count, viability and number of unfertilized oocytes show a similar trend? This would support the author's speculation that hormone-like signal secreted by the sperm supports oocyte maturation and ovulation and that the lower sperm count in mutant hermaphrodites is responsible for increase in number of unfertilized oocytes. This section of the results was also lacking in the Discussion.
7. Figures 4 and 5 show co-localization analysis of SPSP-1 and SPE-11. The image quality appears to be dull and pixilated (at least in my downloaded version). Could the authors use better representative images and insets here? It would also be useful to use monochrome images for the germline images for color blindness.
8. It is interesting to see that in spsp-1 mutants, SPE-11 signal becomes more diffuse. However, this does not explain any of the mutant-related phenotypes. Why do the authors think that the phenotypes seen in spsp-1 mutants are related to SPE-11 mislocalization…."Perhaps embryonic lethality is the result of some yet unknown function with SPE-11 after fertilization"? Do the authors think it unlikely that SPSP-1 has some other independent role? Can the authors work on making this section of the Discussion more readable?

Reviewer 2

Comments for the author

This study investigates SPSP-1, a binding partner of the SPE-11 protein, and its effects on worm fecundity. The authors report that the null mutation in the spsp-1 gene results in decreased sperm levels as well as causing an embryonic lethality phenotype. The writing, structure and scientific accuracy is mostly clear, however, further explanation is needed for the methods and results sections.

Major points:
1. In line 242, the authors mention that they do not observe any changes in the germ line size. Relevant methods and analyses should be added. Additionally, the paper would benefit from including the related image and bar graph quantification to the Supplementary section.
2. The authors have shown that the spsp-1 mutations lead to embryonic lethality. However, it will

© 2026. Published by The Company of Biologists under the terms of the Creative Commons Attribution License (https://creativecommons.org/licenses/by/4.0/).

be helpful to collect embryos of these worms by either dissecting (early-stage embryos) or from plates washing with M9, several centrifugations at low g and additional washes with M9 for isolating embryos from bacteria (late-stage embryos), and image them under the microscope with a 63× objective to quantify the fraction of embryos that appear normal or abnormal. This method will allow the authors to distinguish embryos based on their developmental stages.

Minor points:
1. I would recommend adding how many biological replicates were done to the methods section of brood assays.

Reviewer's Responses to Questions

Experimental quality
Are experiments performed using appropriate methods that will answer the question (or test the hypothesis or support the observations) posed by the authors? Is the right tool used for the job?
If 'No', please indicate reasons in Comments for Author box below.
Reviewer #1:
- Yes

Reviewer #2:
- Yes

Were the data analyzed using appropriate statistical tests?
If 'No', please indicate reasons in Comments for Author box below.
Reviewer #1:
- Yes

Reviewer #2:
- Yes

Reproducibility
Were experiments performed using adequate number of biological replicates?
If 'No', please indicate reasons in Comments for Author box below.
Reviewer #1:
- Yes

Reviewer #2:
- Yes

Is there sufficient raw data to assess the rigor of the analysis?
If 'No', please indicate reasons in Comments for Author box below.
Reviewer #1:
- Yes

Reviewer #2:
- Yes

Does the methods section provide sufficient detail to permit reproducibility?
If 'No', please indicate reasons in Comments for Author box below.
Reviewer #1:
- Yes

Reviewer #2:
- Yes

© 2026. Published by The Company of Biologists under the terms of the Creative Commons Attribution License (https://creativecommons.org/licenses/by/4.0/).

Completeness
Are the manuscript's conclusions supported by the data?
If 'No', please indicate reasons in Comments for Author box below.
Reviewer #1:
- Yes

Reviewer #2:
- Yes

Scholarship
Do the authors cite and discuss the merits of data that would argue for and against their conclusion?
If 'No', please indicate reasons in Comments for Author box below.
Reviewer #1:
- Yes

Reviewer #2:
- Yes

Does the manuscript title & abstract accurately reflect the contents of the manuscript, without hyperbole?
If 'No', please indicate reasons in Comments for Author box below.
Reviewer #1:
- Yes

Reviewer #2:
- Yes

First revision

Author response to reviewers' comments

[Dear Dr. Koushika,

Thank you for the quick reviews of our paper. We would like to thank the reviewers for their thoughtful comments and feedback on this manuscript. We have carefully considered each comment from both reviewers. Please find attached our revised manuscript bio.062634. Below we address the reviewers' comments:

Reviewer 1 Comments:

1. Given that the sperm count is lower at 6h post L4, but not at 24h post L4, could the authors include day-wise embryonic viability and day-wise brood-size trend?
Thank you for the suggestion. We have now included a new figure, Supplemental Figure 3, to show day-wise brood sizes and embryonic viability. We have also added the following text to the manuscript:
- "To determine if the increased number of oocytes laid by the *spsp-1* mutant hermaphrodites correlated with the decreased brood size of the mutants, we analyzed this same data set for the average number of progeny laid and embryonic viability of the progeny. The brood size over a 72-h period reflected the same phenotypes we observed in Figure 1; both *spsp-1(syb2478)* and *spsp-1(syb2479)* had reduced brood sizes compared to N2 (**Fig. S3A**, N2= 228.8 ± 10.0, *spsp-1(syb2478)*= 186.1 ± 8.5, *spsp-1(syb2479)*= 176.5 ± 6.9). When the brood sizes were analyzed by day, there was not a significant difference in

© 2026. Published by The Company of Biologists under the terms of the Creative Commons Attribution License (https://creativecommons.org/licenses/by/4.0/).

the number of progeny on day one, but a reduced number of progeny were observed on day two and three (**Fig. S3B**). These data are consistent with the reduced number of hermaphrodite self-sperm produced by *spsp-1(syb2478)* and *spsp-1(syb2479)* (**Fig. 2C**). As the hermaphrodite self-sperm are depleted through fertilization, overtime, fewer progeny are produced."

- "As we also found that *spsp-1(syb2478)* and *spsp-1(syb2479)* had a subtle decrease in embryonic viability, we asked if the increased number of oocytes laid correlated with embryonic viability. Embryonic viability over the 72-h period was reduced for both *spsp-1(syb2478)* and *spsp-1(syb2479)* compared to N2 (**Fig. S3C**, N2= 97.0% ± 1.1., *spsp-1(syb2478)*= 86.9% ± 3.2, *spsp-1(syb2479)*=
80.4%± 2/5). The percent embryonic viability by day showed that embryonic viability was reduced for each day (**Fig. S3D**). As dead embryos were observed each day with the numbers proportional to the number of embryos laid per day, we conclude that the defects causing embryonic lethality remains consistent over the reproductive lifespan of the hermaphrodite."

2. Surprised to see that reciprocal crosses produce similar brood sizes. Could the authors also include embryonic viability/lethality in these experiments? The conclusion at line 129 feels vague. Could the authors clarify this further in the Discussion section and improve the writing of this section in the Results?

We have now included graphs showing percent embryonic viability from the reciprocal mating experiments (Supplemental Figure 2). Additional text has been added to the results and discussion sections of the manuscript:

- ***Results:*** "We also examined the progeny of the reciprocal matings for embryonic lethality. The slight reduction in embryonic viability of the *spsp-1* mutants was restored when wild-type sperm were used for fertilization (**Fig. S2**, N2=96.5% ± 0.6, *spsp-1(syb2478)*=94.6 ± 1.2, *spsp-1(syb2479)*=95.4 ± 1.0). These results suggest that the reduction in brood and embryonic viability could stem from a sperm defect. When *spsp-1* mutant males were crossed to *fog-2(oz40)* control females, embryonic viability was very slightly yet significantly reduced when *spsp-1(syb2478)* mutants were the sire sires (**Fig. S2B**, N2=98.6% ± 0.2, *spsp-1(syb2478)*=96.1% ± 0.5). There was not a statistically significant difference in embryonic viability between N2 and *spsp-1(syb2479)* sires (**Fig. S2B**, N2=98.6% ± 0.2, *spsp-1(syb2479)*=98.3% ± 0.3). These results indicate that *spsp-1* is not required for male fertility. However, *spsp-1* is required for full hermaphrodite self-fertility."
- ***Discussion:*** "We also found that embryonic lethality is rescued in the reciprocal mating crosses (**Fig. S2**)."…"Male-derived sperm are deposited into the uterus and must travel to the spermatheca, the site of fertilization (L'Hernault 2006). Although we did not observe defects in sperm morphology or sperm activation (**Fig. 3C**), if a small proportion of the male sperm have defects, the sperm without defects would be more competitive, reach the spermatheca and available for fertilization. Future experiments will be important to decipher the multiple roles of the novel SPSP-1 protein."

3. The hermaphrodite sperm count is found to be less at 6h post L4. Could the authors conduct this analysis on mutant males from the reciprocal cross experiments? Can the authors comment on the kinetics of spermatogenesis, cell division, or sperm migration in the Discussion?

It would be interesting to find if males also produce fewer sperm. However, these experiments would be challenging and difficult to observe a difference if the defect in sperm production is subtle. We have included a new section in the Discussion:

- "SPSP-1 is found in both hermaphrodite and male spermatogenic germ lines (**Fig. 4**), so why do we not observe a defect in male fertility in the *spsp-1* mutants? Unlike hermaphrodites, which produce a finite number of sperm during the last larval stage (L4), male *C. elegans* continually produce sperm throughout their entire life (Singson 2001, Ward & Carrel 1979). Thus, males make excess sperm compared to hermaphrodites and a slight decrease in sperm production, as found in *spsp-1* mutant hermaphrodites, could be missed."

4. In Figure 3C, can the percentage of sperm activation be included?

© 2026. Published by The Company of Biologists under the terms of the Creative Commons Attribution License (https://creativecommons.org/licenses/by/4.0/).

Thank you for the great suggestion! We have included the percentage of activated sperm in the figure for each sample. We have also added the following text to the figure legend:

- (C) *In vitro* pronase-activated N2 and *spsp-1Δ* male spermatozoa. Sperm sample sizes (inactivated): N2 N=139, *spsp-1(syb2478Δ)* N=125, and *spsp-1(syb2479Δ)* N=102. Sperm sample sizes (activated): N2 N=128, *spsp-1(syb2478Δ)* N=129, and *spsp-1(syb2479Δ)* N=104.Scale bar= 5 µm. (D) Hermaphrodite self-sperm activated *in vivo*. Sperm sample sizes: N2 N=149, *spsp-1(syb2478Δ)* N=100, and *spsp-1(syb2479Δ)* N=49. Scale bar= 5 µm. The percentage of activated sperm is indicated in the bottom left corner. Sperm activation data was collected from at least three replicates.

5. In Figures 3A and 3B, I believe the Y-axis should be the average of unfertilized oocytes laid not total oocytes?

We have fixed the y-axes of Figures 3A and 3B and updated the related figure legends.

6. Figures 4 and 5 show co-localization analysis of SPSP-1. It would be useful to use monochrome images for the germline images for color blindness.

For Figure 4A-D we have changed all the images of single colors to black and white leaving the merged panels in color. For Figure 5 we changed the single-color images in panels A and C to black and white with the merge panels in color. However, for 5B and 5D we opted to leave all panels in color in order to highlight the co-localization of SPSP-1 and SPE-11. However, the color scheme, green and magenta, was chosen as color blind friendly according to the American Society for Cell Biology Diversity, Equity, and Inclusion, Science (https://www.ascb.org/diversity-equity-and-inclusion/how-to-make-scientific-figures-accessible-to-readers-with-color-blindness/).

7. Why do the authors think that the phenotypes seen in *spsp-1* mutants are related to SPE-11 mislocalization? Do the authors think it unlikely that SPSP-1 has some other independent role?

At this time, we do not know if SPSP-1 and SPE-11 have dependent or independent functions. Although we discovered SPSP-1 through a genetic interaction with SPE-11 and they co-localize, it is entirely possible and maybe even likely based on their distinct phenotypes, that they have independent functions in spermatogenesis and embryogenesis. We have added the following sentence to the Discussion to clarify this point.

- “Taken together, SPSP-1 does not appear to be involved in egg activation like SPE-11 and may have functions independent of SPE-11.”

Reviewer 2 Comments:

1. In line 242, the authors mention they do not observe any changes in the germ line size. Relevant methods and analyses should be added. Additionally, the paper would benefit from including the related image and bar graph quantification to the Supplementary section.

We did not make any direct measurements of the germ line and we apologize if we overstated our observations of the germ lines. We have changed the manuscript text to: “However, neither SPSP-1 nor SPE-11 are present in the proliferative zone, suggesting that these proteins are unlikely to play a role in mitotic proliferation. Nevertheless, it remains possible that SPSP-1 and SPE-11 are present at levels below the detection threshold of our assays.”

2. It would be helpful to collect dead embryos from the *spsp-1* mutant worms to distinguish embryos based on their developmental stages.

Thank you for the suggestion. We have collected dead embryos from *spsp-1(Δ)* mutants raised at 24°C and DAPI stained them to determine how far they develop before dying. *spsp-1(Δ)* dead embryos appear to arrest during grastrulation while some embryos may develop to the elongation stage. The following text has been added to the materials and methods, results, and discussion.

- *Materials and Methods:* “For sperm quantification assays five to ten animals were picked onto a coverslip containing 5 µL M9 buffer. For imaging of *spsp-1(Δ)* dead embryos, dead

© 2026. Published by The Company of Biologists under the terms of the Creative Commons Attribution License (https://creativecommons.org/licenses/by/4.0/).

embryos were picked from agar plates and placed onto a coverslip with 5 µL M9 buffer.”

- *Results:* To determine the developmental stage the dead embryos reached, we imaged embryos by DIC and DAPI staining. As only ~20% of *spsp-1* mutant embryos are nonviable, we recovered dead embryos from plates incubated at 24°C for 48 h. Embryos from both *spsp-1(syb2478Δ)* and *spsp-1(syb2479Δ)* were able to undergo several rounds of cell division. Most of the embryos appear to have arrested during gastrulation (**Fig. S1D**). Some embryos have creases that resemble attempted folding of the embryo during the elongation stage (**Fig. S1D**, Example 2). However, these embryos do not resemble the characteristic morphologies of the elongation stage (e.g., comma, 1.5-fold, 2-fold, or 3-fold) (Hall et al., 2017). From these observations we conclude that *spsp-1(syb2478Δ)* and *spsp-1(syb2479Δ)* embryos arrest between gastrulation and elongation, suggesting that SPSP-1 is required for embryonic development past gastrulation.
- *Discussion:* Furthermore, unlike *spe-11* mutants *spsp-1(Δ)* embryos have intact, impermeable eggshells with most embryos arresting prior to elongation (**Fig. S1D and S5**). *spe-11* mutant embryos make defective eggshells, fail to complete oocyte meiosis and to produce polar bodies (Hill et al., 1989; Johnston et al., 2010; L’Hernault et al., 1988; McNally and McNally, 2005; Sato and Sato, 2011).

3. Adding how many biological replicates were done to the methods section of brood assays.

Thank you for bringing this omission to our attention. We have added the following text to the methods section:
“Data in graphs represent three biological replicates with at least 10 hermaphrodites for each replicate.”

Second decision letter

MS ID#: bio.062634R1

MS Title: SPSP-1 is a sperm protein required for C. elegans hermaphrodite sperm production and embryogenesis

Authors: Aimee Jaramillo-Lambert, Darline Murat, Margaret Nagorski, Ji Kent Kwah, Shannon Pfeiffer, Nancy Duker and Frank Döring

I am happy to tell you that your manuscript has been accepted for publication in Biology Open, pending our standard publication integrity checks. It was accepted on 4th June 2026.

© 2026. Published by The Company of Biologists under the terms of the Creative Commons Attribution License (https://creativecommons.org/licenses/by/4.0/).
